# ^13^C CPMAS NMR as a Tool for Full Structural Description of 2-Phenyl Substituted Imidazoles That Overcomes the Effects of Fast Tautomerization

**DOI:** 10.3390/molecules25173770

**Published:** 2020-08-19

**Authors:** Nikola Burdzhiev, Anife Ahmedova, Boris Borrisov, Robert Graf

**Affiliations:** 1 Faculty of Chemistry and Pharmacy, Sofia University, 1, J. Bourchier blvd., Sofia 1164, Bulgaria; bborrisovchem@gmail.com; 2 Max Planck Institute for Polymer Research, Ackermannweg 10, 55128 Mainz, Germany; graf@mpip-mainz.mpg.de

**Keywords:** solid-state NMR, 2-phenylimidazolecarbaldehydes, tautomerization, GIAO-DFT calculations, synthesis

## Abstract

Tautomerization of 2-phenylimidazolecarbaldehydes has not been studied in detail so far, although this process is a well-known phenomenon for imidazole derivatives. That is why we focus our study on a series of 2-phenylimidazolecarbaldehydes and their parent alcohols that were synthesized and studied by detailed ^1^H and ^13^C NMR in solution and in the solid state. The apparent problem is that the fast tautomerization impedes the full structural description of the compounds by conventional ^13^C NMR measurements. Indeed, the ^13^C NMR spectra in solution exhibit poor resolution, and in most cases, signals from the imidazole ring are not detectable. To avoid this problem, we used ^13^C CP-MAS NMR as an alternative spectroscopic method for unambiguous spectroscopic characterization of the studied series of 2-phenylimidazoles. The data were analyzed in combination with quantum chemical DFT-GIAO methods by considering the tautomerization process and the intermolecular interactions. The DFT (B3LYP/6-31G(d,p)) calculations allowed to identify and suggest the preferred tautomer in the gas phase and in DMSO solvent, which for alcohols are (2-phenyl-1*H*-imidazol-4-yl)methanol and its analogs, and for the aldehydes are the 2-phenyl-1*H*-imidazole-5-carbaldehydes. The gas-phase calculated energy differences between the two possible tautomeric forms are in the range 0.645–1.415 kcal/mol for the alcohols and 2.510–3.059 kcal/mol for the aldehydes. In the DMSO solvent, however, for all compounds, the calculated energy differences go below 1.20 kcal/mol. These data suggest that both tautomeric forms of the studied 2-phenylimidazoles can be present in solution at room temperature. Our data from detailed 2D NMR measurements in the solid state (^1^H-^13^C HETCOR and ^1^H-^1^H double-quantum coherence MAS NMR) suggested that also in the solid state both tautomers coexist in different crystalline domains. This fact does not obscure the ^13^C CP-MAS NMR spectra of the studied 2-phenyl substituted imidazoles and suggests this spectroscopic method as a powerful tool for a complete structural description of tautomeric systems with aromatic conjugation.

## 1. Introduction

Tautormerization in imidazoles is a well-known phenomenon and the effect of different substituents on stabilizing either of the two possible tautomers is very well studied [1,2,3]. Due to the rapid tautomerism of unsymmetrically substituted imidazoles with a ring *N*-hydrogen, these compounds mostly exist as tautomeric pairs that are inseparable [1,2]. In many cases, the rate of the switching between the two tautomeric forms is slow compared with the NMR time scale [4] and leads to distinct chemical shifts for the ^1^H signals of the tautomers [2]. This allows for studying conveniently the tautomerization processes in imidazoles by ^1^H-NMR spectroscopy in solutions [5]. Nevertheless, in many recent studies on variously substituted imidazoles, the focus is on their potential applicability rather than their tautomerism. As an important heterocyclic moiety, the imidazole ring could be found in many natural products, pharmaceutical compounds, functional materials [6,7] (dyes, optoelectronics [8]). Besides, the diversity of biological activities of imidazole derivatives ranges from antibacterial, antifungal [9], anti-inflammatory, antitumor, and antimalarial activity [10] to monoamine oxidase inhibition [11] and many more [12,13], including the biological activity of metal complexes of some *N*-heterocyclic carbenes derived from imidazoles [14,15].

The 2-phenylimidazoles, for example, are mostly studied for their biological activity, and often the structural characterizations are scarce [13]. One major reason in some of the cases could be the fast tautomerization of the imidazole ring leading to very broad and weak ^13^C-NMR signals in the solution NMR spectra. The carbon atoms in direct proximity to the tautomerizing sites of the molecule are most affected and their signals are usually not observed in conventional ^13^C-NMR spectra. Therefore, the presentation of ^13^C-NMR spectra of a given compound is sometimes omitted in literature [16,17]. In other cases, aiming at higher precision, significantly longer time for acquisition has been sacrificed to make the missing signals appear acceptably well, as can be seen in the Supplementary Materials of some papers [18,19]. Another way to overcome this phenomenon is to perform variable temperature NMR measurements. This approach, however, is limited by phase transitions of the used solvent and the thermal stability of the analyzed compounds. Solid-state NMR spectroscopy, in particular ^13^C CP-MAS (cross-polarization magic angle spinning) NMR methods, has been successfully used for complete structural description of various tautomeric compounds [20,21,22,23] and even for their metal complexes [24,25,26]. In most cases, the experimental data are correlated with theoretical calculations, usually taking into account both the molecular forms and the media effects on tautomerism—either as a polarity or discrete intermolecular interactions [27]. The advancement in quantum chemical methods proved to provide reliable benchmark for correct description of the nuclear shielding in the molecules and for outlining the main effects of the intramolecular changes or the intermolecular interactions [28].

**Figure 1:**
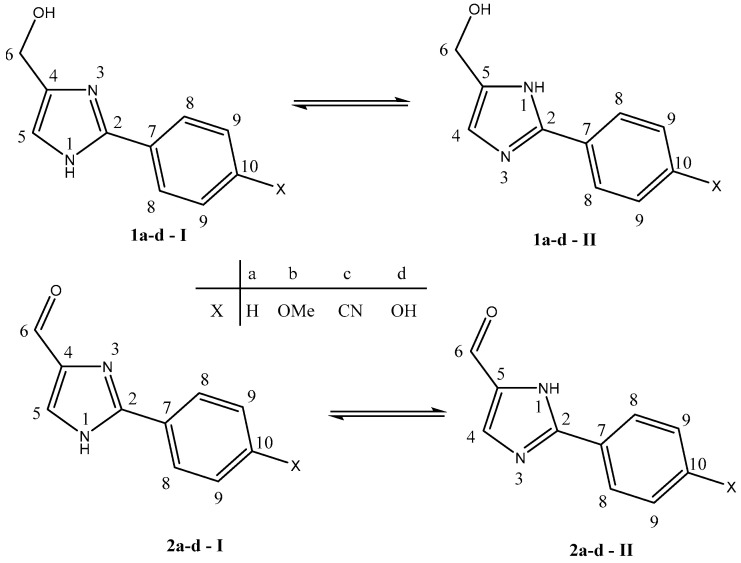
Molecular structures of the studied 2-phenyl substituted imidazoles: **1a**–**d—**(2-phenyl-1*H*-imidazol-4(5)-yl)methanols and **2a**–**d—**2-phenyl-1*H*-imidazole-4(5)-carbaldehydes. Both tautomeric forms (**I** and **II**) are depicted along with the atoms numbering used in the chemical shift assignments of the NMR signals. (Although the numbering is not fully in line with IUPAC guidelines, it is used here for convenience).

Herein, we focus our study on 2-phenylimidazolecarbaldehydes, some of which had previously been exploited as synthons for synthesis of Schiff bases [29,30] or various biologically active molecules [13], but their structure had scarcely been characterized. To the best of our knowledge, there is no crystallographic data reported on 2-phenylimidazolecarbaldehydes and the corresponding methanols, and the available structural descriptions are based solely on ^1^H-NMR spectra, without ^13^C-NMR measurements and complete assignments. The reason, as it appears, is that the ^13^C-NMR in solution is largely obscured by the fast tautomerism in the imidazole ring. Despite the availability of several synthetic methods for imidazoles in general, the alternations in solubility of the compounds, depending on the used starting aldehyde, greatly affects the overall yield. In this work, we optimized the synthetic procedures to obtain the target 2-phenyl imidazole-carbaldehydes and the corresponding alcohols. Further on, we used NMR spectroscopy in solution (DMSO-*d*_6_) and in the solid state to study experimentally the tautomerism of a series of 2-phenylimidazoles, namely (2-phenyl-1*H*-imidazol-4(5)-yl)methanols (**1a**–**c**) and 2-phenyl-1*H*-imidazole-4(5)-carbaldehydes (**2a**–**d**), which have different substituents in the *para*-position of the benzene ring (Figure 1). Quantum chemical calculations were performed to estimate the relative stabilities of the possible tautomers and rotamers of all compounds. For the two most stable tautomers of all compounds, the nuclear magnetic shieldings have been calculated and were correlated with the experimental data.

## 2. Results

### 2.1. Synthesis and NMR Measurements in Solution

The synthesis was achieved by following and, depending on the type of the substituent, significantly improving the existing protocols. For the synthesis of the imidazole ring, we applied the Weidenhagen reaction [31], according to the experimental procedure described by Baldwin et al. [32] and introducing some modifications at the work-up stage. The aldehydes **2a**–**c** were obtained from the corresponding alcohols (**1a**–**c**) by mild oxidation with BaMnO4 that has been freshly synthesized following a published procedure [33]. The aldehyde **2d** was obtained from **2b** by heating for 15 h at 100 °C in 48% HBr, and therefore the corresponding methanol derivative **1d** was not studied experimentally. All compounds were characterized by IR spectroscopy, ^1^H-NMR, and ^13^C-NMR measurements in DMSO-*d*_6_ solution and in the solid state.

The compounds depicted in Figure 1 exhibit fast tautomeric transformation of the imidazole ring in solution, which could be clearly demonstrated by the ^1^H-NMR measurements in DMSO-*d*_6_. Note that in the tautomeric form **I** the C-H hydrogen atom is localized at the C5 carbon atom, whereas in the tautomeric form **II** the numbering is changed to C4 due to alternation of the atom numbering in the imidazole ring that is associated with the changed position of the N-H hydrogen during tautomerization. For most of the studied aldehydes (**2a**–**d**), two distinct signals for the N-H proton could be identified, as could be seen in Table 1 that summarizes the ^1^H-NMR data. Only in the case of the benzonitrile derivative **2c**, just one signal could be observed for an N-H proton, but for its alcohol analog **1c**, two signals at 12.71 and 12.85 ppm could be distinguished (Figures S16 and S37 for **1c** and **2c**, respectively).

**Figure 2:**
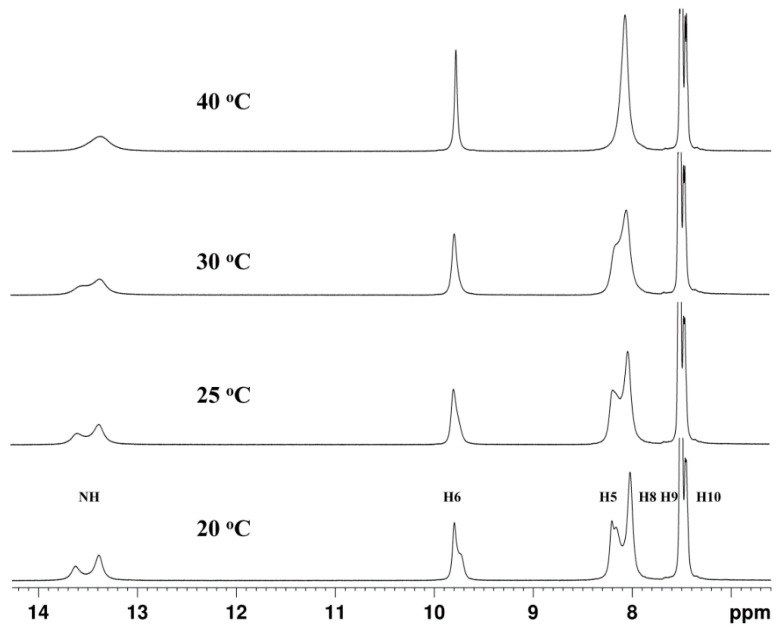
Temperature-dependent ^1^H-NMR spectra (DMSO-*d*_6_) of compound **2a**.

For compounds **1c** and **2a**, the ^1^H-NMR signals of the N-H proton were sufficiently intensive to perform temperature-dependent measurements up to 40 °C (Figure 2 for **2a** and Figure S17 for **1c**). Thereby, the ratio of the existing tautomers at temperature of 25 °C was estimated to be around 40/60, measured by the intensities of the signals of N-H protons for both tautomeric forms and processed with deconvolution analysis. However, the ^1^H-NMR spectra were not sufficient to assign each of the signals to one particular tautomer. The variable temperature experiments showed that the rate of the tautomerism depends also on the concentration of the solution—the less concentrated the solutions, the better the resolution of the ^1^H spectra at lower temperature (Figure S47). This could be associated with the different types of intermolecular interactions that govern the tautomerism in the studied 2-phenyl imidazoles, which make the tautomerization process multifactorial. Due to the very limited solubility of the compounds, DMSO was the only suitable choice for solvent. Although other solvents may allow recording spectra at lower temperatures, the change of the solvent will add another factor that may alter the tautomerization process, making the data case-specific and with less practical importance.

The ^13^C-NMR spectra in solution proved unreliable due to the inability to register the C-atoms from the imidazole ring for all studied compounds. In all cases, at least two signals were missing in these spectra even if highly extended transient numbers were used (with number of scans of 1024 or 2048, Figures S29 and S39 for **2b** and **2c**, respectively). These observations are partly in accordance with earlier reported data on 2-phenyl imidazoles where either no or incomplete ^13^C-NMR assignments are provided [16,17]. This incompleteness could be explained with the time scale of tautomeric exchange in solutions, which might be comparable with that of the ^13^C-NMR experiment.

### 2.2. Solid-State NMR Measurements

**Figure 3:**
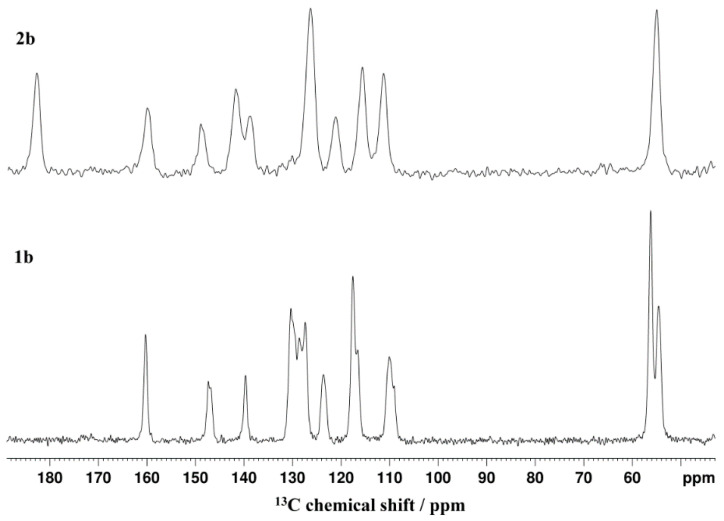
^13^C CP-MAS NMR spectra of compounds **1b** and **2b**. For signal assignments see Table 2 and Table 3.

To avoid the described incompleteness of the ^13^C-NMR in solution, we performed ^13^C CP-MAS NMR measurements in the solid state. For all studied compounds, the ^13^C CP-MAS NMR spectra clearly indicated strong signals for each of the carbon atoms, as shown in the Supplementary Materials. At first glance, the solid materials do not show evidence for presence of two different tautomeric forms, except for sample **1b**. The ^13^C CP-MAS spectrum of **1b** is shown in Figure 3 in comparison with the spectrum of the corresponding aldehyde **2b**. In the case of compound **1b**, where the CP-MAS signals are particularly sharp, a distinct splitting of the signals at 147 ppm and 110 ppm is observed. We are aware that the crystallization of molecules can lead to splitting of NMR signals by distinguishing magnetically inequivalent but chemically equivalent sites if there is more than one molecule in the crystallographic unit cell or if the molecular symmetry is broken by the symmetry of the crystallographic lattice. However, both reasons can be excluded in our systems on the basis of the additional NMR experiments that we performed in the solid state. Cross-polarization/polarization-inversion (CPPI) measurements for compound **1b** allowed for clear distinction of the quaternary C-atoms in the solid state and facilitated the signal assignments (Figures S6, S14 and S26 for **1a**, **1b**, and **2a**, respectively). The CPPI spectrum of **1b** allowed us to distinguish the methoxy and alkoxy carbons that exhibit chemical shifts of 55.8 and 54.2 ppm, respectively. Most importantly, the CPPI measurement showed that an additional signal of a quaternary C-atom with a chemical shift of 130.0 ppm is hidden under the signals of the CH carbons of the phenyl ring (Figure S14). Thus, five quaternary carbon atoms were detected in the aromatic region instead of the expected four quaternary carbons for **1b** (two from the benzene and two from the imidazole ring). The chemical shifts of the quaternary carbons approximate to the values of the calculated chemical shifts for both tautomeric forms, as shown in Table 2. This was the first suggestion that in sample **1b** both tautomers are present in the solid state, which motivated our more detailed solid state NMR studies on all other compounds as well.

**Figure 4:**
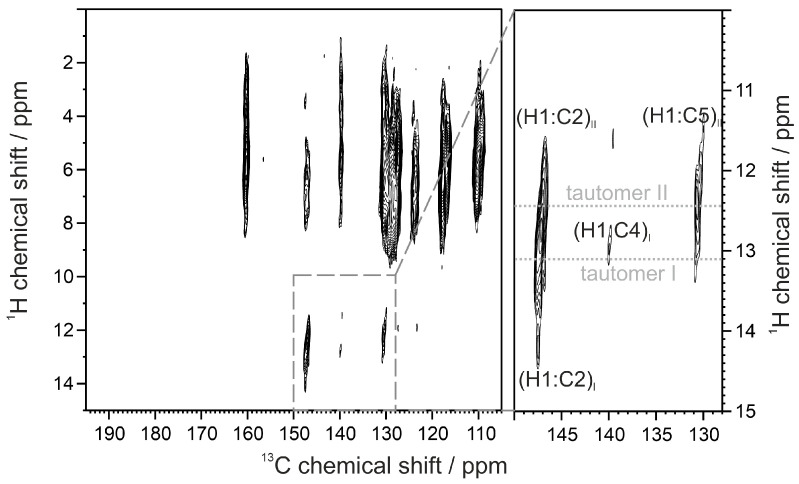
^1^H-^13^C CP-MAS correlation spectrum of compound **1b**. Right block shows magnified view of the selected area in the correlation spectrum.

An additional proof for the co-existence of both tautomeric forms in the solid state is provided by the ^1^H-^13^C CP-MAS correlation spectrum (HETCOR) of compound **1b** (Figure 4), where two distinct correlation patterns for both NH sites are seen. These are indicated in the magnified part of the correlation spectrum (right block in Figure 4) as correlations from tautomer **I** (at 13.1 ppm for NH) and tautomer **II** (at 12.4 ppm for NH). The former NH site is adjacent to only one quaternary carbon atom, which is C2 in tautomer **I** (Figure 1), whereas the NH site in tautomer **II** is adjacent to two quaternary carbon atoms (C2 and C5). These three correlation signals are well seen in the spectrum of **1b**, although the signals for the H1-C2 correlations from tautomer **I**—(H1:C2)_I_ and from tautomer **II**—(H1:C2)_II_ slightly overlap due to proximity of their ^13^C-chemical shifts. We like to point out that CP-MAS correlation signals are through space correlations via hetero-nuclear dipolar couplings and that correlations to protonated ^13^C sites are in most cases truncated by dominating hetero-nuclear dipolar couplings of the covalently bound hydrogen. The (H1:C4)_I_ correlation signal from tautomer **I** is very weak because the atoms are not adjacent, but becomes detectable when the intensity of the signals is enhanced, as is the case depicted in Figure 4.

**Figure 5:**
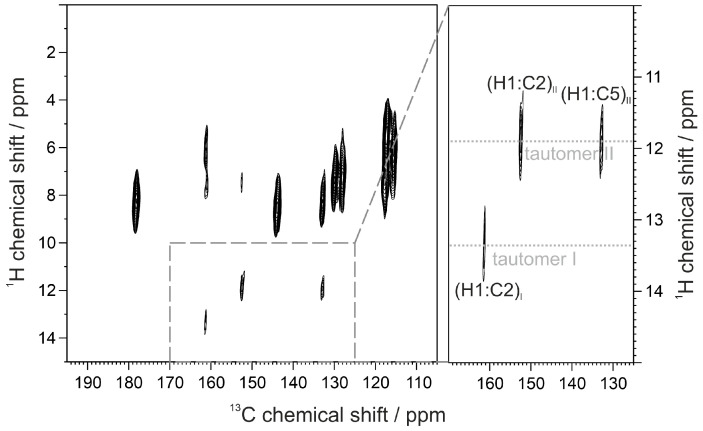
^1^H-^13^C CP-MAS correlation spectrum of compound **2d**. Right block shows magnified view of the selected area in the correlation spectrum.

It should be noted that the presence of both tautomeric forms in the solid material is observable for most samples (Figures S33 and S42 for **2b** and **2c**, respectively). The clear distinction of the two NH chemical shifts and ^1^H-^13^C CP-MAS correlation signals—to one (in tautomer **I**) or two (in tautomer **II**) neighboring quaternary carbons—are most prominent in the case of compound **2d** (Figure 5). In this case, both the ^1^H and the ^13^C chemical shifts of NH and the neighboring quaternary carbon atoms from each of the tautomeric forms are well separated and exhibit unambiguous correlation spectrum. The magnified part of the correlation spectrum (right block in Figure 5) indicated the two distinct correlation patterns, at 13.3 ppm for NH from tautomer **I** and at 11.9 ppm for NH from tautomer **II**.

Moreover, similarly to the CPPI spectra, the ^1^H-^13^C CP-MAS correlation spectra made it possible to identify overlapping signals in the ^13^C CP-MAS NMR spectra that are summarized in Table 2 and Table 3. This also allowed, in some cases, to assign some of the signals to either tautomer **I** or tautomer **II**. For example, the signals of the quaternary C-atoms of **1b** at 139.3 and 130.0 ppm became visible from the CPPI spectra and were assigned to C4 in tautomer **I** and to C5 in tautomer **II**, respectively, with the help of the ^1^H-^13^C CP-MAS correlation spectrum in Figure 4. Similarly, the signals at 147.0 and 131.2 ppm could be identified and associated with the quaternary C2 and C5 atoms of tautomer **II** of compound **2c** from its ^1^H-^13^C CP-MAS correlation spectrum (see Figure S42). For compound **2d,** the correlation spectrum in Figure 5 revealed the presence of additional signal at 161.0 ppm for quaternary C-atom, which correlates with the NH proton in tautomer **I**, and was covered by the signal of the C10 atom seen at 161.2 ppm in the ^13^C CP-MAS NMR spectrum. Thereby, the signal at 161.0 ppm was assigned to C2-atom of tautomer **I**, whereas the signals for quaternary carbons at 152.4 and 132.9 ppm were assigned to C2 and C5 atoms in tautomer **II** of **2d**, respectively.

**Figure 6:**
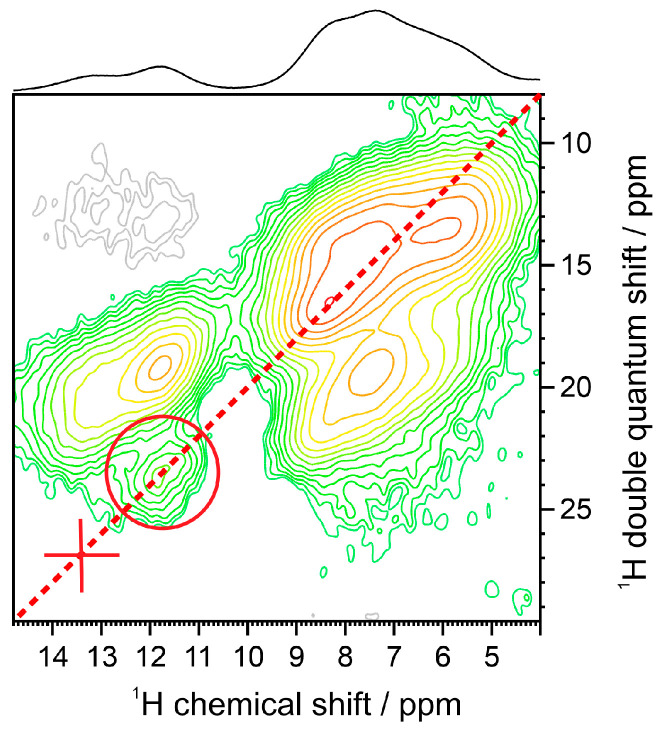
Double quantum-single quantum ^1^H-^1^H correlation spectrum of **2d** recorded at 25 kHz MAS, and 2 rotor periods double-quantum recoupling using the BABA-xy16 pulse sequence.

For selected cases (**2b**, **2c**, and **2d**), ^1^H double quantum coherence experiments have been performed in an attempt to identify the arrangement of the molecules in the crystal lattice. In the case of **2d** (Figure 6, and for **2c** Figures S43 and S44), the correlation peak at 11.9 ppm in the chemical shift dimension and 23.8 ppm in the double quantum dimension (red circle) indicates spatial proximity between the N-H protons of the molecules of tautomer **II** within less than 4 Å. The corresponding correlation signal for form **I**, which should be observed at 13.4 ppm in the chemical shift dimension and 26.8 ppm in the double quantum dimension (red cross), is not detected. This demonstrates that the two tautomeric forms of **2d** crystallize in different crystalline domains that stabilize the different tautomeric forms and allow for tautomeric exchange only within the domain. Similar are the observations for the benzonitrile derivative **2c**. It is worth noting that from our numerous attempts to obtain good quality single crystals from all studied compounds, for compound **2c** we obtain visibly different crystalline phases that slightly differ in color, however, no single crystals (microscope image Figure S56).

The results from the ^13^C CP-MAS NMR measurements are summarized in Table 2 and Table 3 for the studied alcohols (**1a**–**c**) and carbaldehydes (**2a**–**d**), respectively. The given assignment of the registered signals was facilitated by ^1^H-^13^C CP-MAS correlation experiments with different contact times and by quantum chemical calculations. Identification of the quaternary carbon atoms by the CPPI MAS NMR experiments was helpful for the assignment but not sufficient to distinguish the two tautomers. In all compounds from the studied series, signals for all quaternary C-atoms were identified. The two quaternary carbon atoms from the imidazole ring of the alcohols (**1a**–**c**) exhibit chemical shifts in the range of 149–139 ppm, and in the carbaldehydes (**2a**–**d**) from 152 to 132 ppm. Correlation experiments could allow for differentiation of the tautomeric forms only for the non-overlapping signals, but most of their signals in the studied compounds overlap. Only in the already discussed case of **1b**, one additional signal for a quaternary carbon appeared, and a couple of other signals showed splitting. This imposed the necessity to perform quantum chemical calculations of the possible tautomers and rotamers of the studied series of compounds and to estimate the nuclear magnetic shielding for all carbon atoms.

### 2.3. Quantum Chemical Calculations

**Figure 7:**
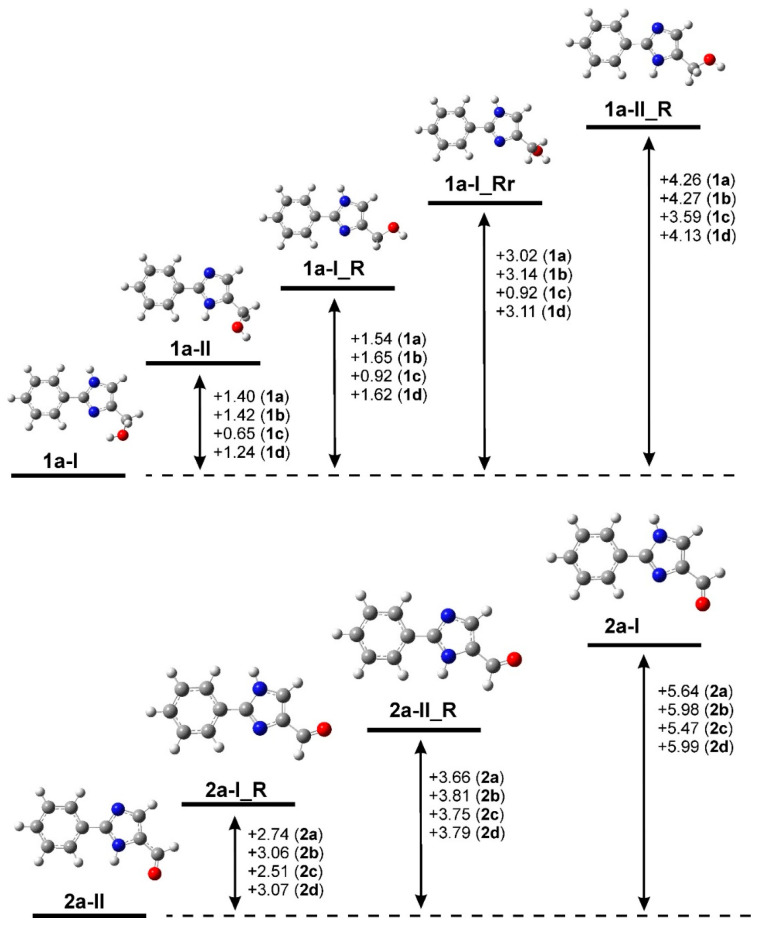
DFT calculated energy differences (in kcal/mol, and corrected for ZPVE) for all optimized (B3LYP-6-31G(d,p) in gas phase) geometries of the possible rotamers (**R** and **Rr**) and tautomers (**I** and **II**) of the 2-substituted imidazoles: (top) **1a**–**d—**(2-phenyl-1*H*-imidazol-4(5)-yl)methanols, structures shown for **1a** only, and (bottom) **2a**–**d—**2-phenyl-1*H*-imidazole-4(5)-carbaldehydes, structures shown for **2a** only.

The geometry optimizations of both tautomers (forms **I** and **II** in Figure 1) and some of their possible rotamers (in total 5 types for the alcohols and 4 types for the aldehydes) were performed in gas phase and in DMSO solvent. The B3LYP functional and the 6-31G(d,p) basis set were used for all types of calculations. For selected compounds, other functionals and broader basis set have been employed but no change in the tendencies was observed. Therefore, we limited the theoretical description to the method B3LYP/6-31G(d,p) which, according to our experience, provides reliable results also for the nuclear shielding by the gauge-independent atomic orbital (GIAO) calculations [24,25,26,34]. The calculated energy differences for all optimized geometries are depicted in Figure 7.

Energetically most stable tautomer for the alcohols is form **I** (with the hydrogen at C5 atom), whereas for the aldehydes **2a**–**d** the most stable tautomer is form **II** (with the hydrogen at C4). The energy difference between the forms **I** and **II** of the alcohols is less than 1.42 kcal/mol, according to the gas phase calculations. The closest in energy are the tautomers of compound **1c** (0.645 kcal/mol). Similar tendency is seen for the aldehydes where the energy difference between the forms **II** and **I_R** ranges from 3.065 (for **2d**) to 2.510 kcal/mol (for **2c**). The obtained energy profile and the large difference in the polarity of some of the forms indicated that the role of the environment may become crucial for defining the preferred forms. Therefore, we also performed calculations in DMSO solvent using the PCM – polarizable continuum model and the same density functional theory (DFT) method.

The calculations in the polar environment of DMSO solvent have shown that the energy profiles for the tautomers/rotamers of all compounds remain the same as the one in the gas phase (Figure 7). The main effect is the significant decrease in the energy difference between forms **II** and **I_R** for the aldehydes, which in the case of **2c** goes down to 0.460 kcal/mol. For all other compounds, the energy differences between the two tautomers go below 1.20 kcal/mol in DMSO. These results agree with the experimental evidences that both tautomers exist in solution, which were obtained from the NMR measurements in DMSO-*d*_6_ solvent. Unfortunately, the solution NMR data were not sufficient to assign the seen signals in the ^1^H NMR spectra to a particular tautomer to allow us to verify the predicted population of the calculated forms. That is why we used the calculations to qualitatively suggest the most stable tautomer for the compounds and to monitor the changes in the ^13^C-NMR shieldings upon tautomerization and intermolecular interactions.

The ultimate goal of the quantum chemical calculations in this study was to estimate the nuclear shielding and the corresponding NMR shifts and to find correlations with the experimental ^13^C CP-MAS NMR spectra of compounds **1a**–**c** and **2a**–**d**. This was performed by the GIAO approach using the same DFT method as for the geometry optimizations. The calculated ^13^C NMR shifts for the two most stable forms of each compound are listed in Table 2 (for **1a**–**d**) and Table 3 (for **2a**–**d**), together with the experimental data that are given in bold. The signals of the quaternary C-atoms have been identified through additional experimental techniques and the corresponding chemical shifts are underlined in the tables.

Close inspection of the experimental and the calculated ^13^C NMR shifts suggests that the two most stable tautomeric forms in the gas phase are observable in the ^13^C CP-MAS NMR spectra. It should be noted, however, that for many C-atoms the calculated NMR shifts from both tautomers almost coincide. For example, the chemical shifts for C2 atoms from both tautomers of the alcohols **1a**–**d** coincide (ca. 139 ppm) and are very close to the value obtained for the C4 atom of form **I** (see Table 2). Moreover, the calculated shifts for C4 and C5 atoms of form **II** are close to one other (in the range 122–127 ppm) and interfere with the signals in the aromatic region from the benzene ring. Similar evidences for significant overlap of the signals in the solid-state NMR spectra were discussed in the previous section. Nevertheless, we can claim that the obtained agreement between the experimental and the calculated chemical shifts is fairly acceptable as general tendencies, based on the experimental data of the two tautomers of compound **1b**. A characteristic tendency seen in the solid-state NMR spectra of compounds **1a**–**c** is that the signals for the C2 and C4 atoms differ no more than 8 ppm (2.3 ppm for **1c** and 7.8 ppm for **1a**). As the calculations suggest, under the chemical shift for C2 can hide both the C2 and C4 signals from form **I**. The calculated values for C5 in forms **I** and **II** is quite distinguishable, however, the value for form **II** may be covered by the signals from the aromatic ring in the experimental spectra.

The GIAO calculations for the aldehydes **2a**–**d** can suggest from the first glance that the tautomeric form observed in their ^13^C CP-MAS NMR spectra is most probably the form **II** (see Figure 1 and Figure 7). However, in these cases also, the calculated chemical shifts for C2 and C4 of form **I_R** almost coincide and are very close to the value of C2 of form **II** (Table 3). On the other hand, the experimentally registered shift for C6 is very characteristic and is closer in value to the calculated ones for form **I_R**. Main tendencies seen from the calculated NMR shifts for forms **II** and **I_R** of the aldehydes (Table 3) can be outlined as follows: (i) for form **I_R** it is characteristic that the chemical shifts for C2 and C4 are very close and almost coincide, whereas the C5 has a chemical shift by ca. 35 ppm lower than C4; (ii) for form **II** the chemical shift for C2 is by ca. 10 ppm higher than the C4 and by ca. 15 ppm higher than the chemical shift of C5. It is evident that the experimental data exhibit a mixture of both tendencies without clear preference to one of them. Here again, some of the calculated values for the C-atoms from the tautomerizing unit are in the range of the signals of the benzene ring, which is the reason they are not observed in the experimental spectra.

In an attempt to monitor the effect of intermolecular interactions on the calculated chemical shifts, we modeled two types of dimers and three types of trimers for the tautomeric forms **II** and **I_R** of compound **1c**. All dimers and trimers were fully optimized and GIAO calculations have been performed at the same level of theory. The main changes observed in the calculated chemical shifts of the dimers and trimers concern the C2 and C4 atoms and only little or no changes are seen in the shielding of C5. Generally, the chemical shifts of C2 and C4 increase when they are involved in intermolecular H-bonding. Given the complicated case of several sets of overlapping signals, as predicted from the gas phase calculations and the experimental NMR data, we concluded that even more elaborate calculations will not help for exact assignment and calibration of the theoretical model.

## 3. Discussion

Although not in the focus of this work, a significant effort was the synthesis of all studied seven 2-phenyl imidazole derivatives, of which only compound **2d** has not been reported so far. Nevertheless, we provide some discussion on the synthetic procedures as well; since our experience showed that general procedures are not working equally well for the differently substituted derivatives. The first synthesis of (2-phenyl-1*H*-imidazol-4(5)-yl)-methanol (**1a**) was described by Lawson in 1957 and is based on a condensation reaction in aqueous ethanol between α-amino-aldehydes, derived from α-amino acids, and methyl or ethyl benzimidates [35]. Later Dziuron and Schunack [36] reported an alternative synthesis of **1a** that involves reacting benziminoethyl ester hydrochloride and 1,3-dihydroxyacetone in liquid ammonia in an autoclave at 70 °C and 20 atm for 5 h, leading to a yield of 73%. In these early reports on the synthesis of **1a,** only melting points and elemental analyses are given. Most recently, Davood et al. [37] used the Weidenhagen imidazole synthesis to prepare **1a** in 40% yield and m.p. 145–147 °C (acetone). Oxidation of **1a** to the carbaldehyde **2a** has been achieved by using MnO_2_ in acetonitrile, but no details are given in the experimental section. The series of imidazole derivatives have been characterized mainly by ^1^H NMR spectra, whereas ^13^C NMR spectral data have been provided only for some of the fully substituted chloroimidazole carbaldehydes. As a modification, Baldwin et al. [32] used methanol during the reaction to avoid additional purification of the complex. However, both methods use H_2_S, which is a toxic gas and is passed through the suspension of the complex in water. We avoided the use of H_2_S by dissolving the complex in hydrochloric acid and adding solid sodium sulfide in small portions. The suspension was filtered and washed with water (small amount) and the pH of the filtrate was adjusted to 9 by solid sodium carbonate to avoid dilution. Although the Weidenhagen reaction is not the best one in terms of final yield, we preferred it because of the ease of the procedure and the availability of the starting materials.

The oxidation of the alcohols **1** to aldehydes **2** can be done by various oxidants. Davood et al. [37] used MnO_2_ but the reaction is too slow and needs large excess of the oxidant. Aldehyde **2a** can be synthesized by oxidation with conc. HNO_3_, according to Paul et al. [38]. The reaction is fast but should be noted that heating for more than 1 h leads to oxidation of the formed aldehyde to a carboxylic acid. This was the best method for the preparation of **2a** from all our synthetic attempts. However, it was not applicable for **2b** due to the presence of the activating methoxy group, which leads to a side reaction of electrophilic substitution. Even in the case of **2c**, the yield was not satisfactory; therefore, we applied BaMnO_4_ as an oxidizing agent. Despite the relatively low yield, the use of BaMnO_4_ was the method of choice for **2c** because the reaction is fast, and the excess of the oxidizing agent is not as large as in the case of MnO_2_. In the case of **2b**, however, better results were achieved by using K_2_CrO_4_ in 30% H_2_SO_4_ for oxidation at room temperature.

**Scheme 1:**
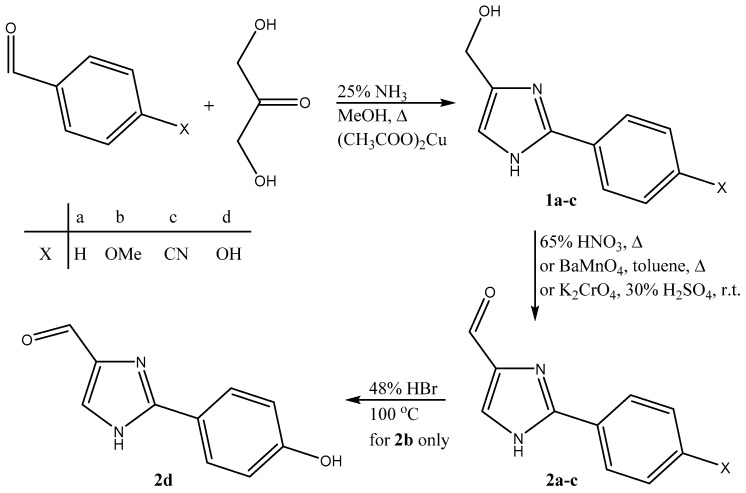
Synthetic procedure for **2a**–**c** and **2d**.

The Weidenhagen reaction from the 4-hydroxybenzaldehyde did not lead to the desired alcohol **1d**. For this reason, we used *p*-anisaldehyde to obtain **1b** and removed the methyl group at a later stage. Heating **1b** in 48% HBr acid removed the methyl group but the hydroxyl group of the alcohol was also substituted with bromine. That is why we first oxidized **1b** to **2b** and then removed the methyl group (Scheme 1). This was achieved by heating **2b** in 48% HBr acid for 15 h, which led to the formation of **2d**. The yield was not very good because these conditions also favor the formation of different polymers. However, due to the presence of a carbonyl group in compound **2b**, most of the known nucleophilic reagents are not applicable because of its additional reactivity. The reaction with BBr_3_ in dichloromethane gave an insoluble complex, which hindered its successful completion. 

For the sake of comparison, we performed ^1^H and ^13^C NMR measurements for the methyl analog of compound **2a**, 2-methyl-1*H*-imidazole-4(5)-carbaldehyde, both in solution and in the solid state (Figures S52–S55). In this case, all signals for the atoms from the imidazole ring were clearly seen both in solution and in the solid state and showed the co-existence of the two possible tautomeric forms. This indicates that, unlike in compound **2a**, the tautomerization of the 2-methyl-1*H*-imidazole-4(5)-carbaldehyde is slower than the NMR time scale. Therefore, we can conclude that the observed phenomenon of very fast tautomerization seen for the 2-phenyl-1*H*-imidazole-4(5)-carbaldehyde and analogs is caused by the presence of the phenyl substituent at the imidazole ring rather than the presence of the alcohol or aldehyde residue. The crystal structure of histaminol was recently solved and it was shown that the compound crystallizes in the form of 5-hydroxyethylimidazole where both N(H)-atoms from the imidazole ring participate in intermolecular hydrogen bonding with the atoms from the OH group [39]. There are very few reports on co-existence of the two tautomers in the crystal structures of imidazoles [40,41]. To the best of our knowledge, there are no evidences for tautomerization in the solid state of 2-substituted imidazoles. Furthermore, no crystal structures of 2-phenyl substituted imidazoles have been reported so far.

It is apparent that the methanols **1a**–**d** have higher flexibility and, therefore, five forms were identified by the theoretical optimizations, whereas the aldehydes have two tautomers with two rotamers for each. Interestingly, the calculations predicted higher stability of form **I** of the alcohols (that is the 5H tautomer, Figure 1), whereas aldehydes are stabilized in the form **II**. For all studied compounds, the DFT optimized most stable tautomeric forms have the CH proton from the imidazole ring parallel to the protons from the adjacent alcohol or aldehyde group. Therefore, different types of NMR correlation experiments have been tested in search of experimental evidences for the existence of these particular tautomeric forms. However, such correlation evidences could not be obtained either because the spectra in solution are too broad and some signals from the imidazole ring are missing or because the presence of intermolecular H-bonding in the solid state obscures the assignment of signals from the ^1^H MAS spectra. Nevertheless, we could find experimental evidences for most of the studied compounds that both tautomeric forms coexist in the solid state from the elaborate NMR measurements that included ^1^H-^13^C CP-MAS correlation experiments with different contact times, CPPI MAS NMR experiments, and ^1^H double quantum coherence experiments, which all complemented the more conventional ^1^H-MAS and ^13^C CP-MAS NMR spectra.

Similarly to the data from the solid-state NMR measurements, our DFT calculations suggested that both tautomeric forms of the studied 2-phenyl substituted imidazoles may coexist in the solution at room temperature, based on the calculated energy differences between all possible tautomers and rotamers. The calculated NMR shifts indicate strong overlap of crucial C-atoms from the imidazole ring either from both possible tautomers or with the signals from the benzene ring. This, on one hand, explains why not all possible signals for both tautomeric forms could be seen in the ^13^C CP-MAS NMR spectra. On the other hand, the calculations support the suggested general conclusion that also in the solid state both tautomeric forms coexist, although only for some atoms additional experiments could identify presence of their signals.

In this work, we have demonstrated that the integrated approach of elaborate NMR experiments in the solid state and theoretical (DFT) calculations could provide reliable structural information on imidazole tautomeric systems conjugated with an aromatic ring despite their fast tautomerization. The calculations helped us to suggest the most stable tautomer for each compound (both in gas phase and in the polar environment of DMSO) and to monitor the changes in the ^13^C-NMR shieldings upon tautomerization and intermolecular interactions. 

## 4. Materials and Methods

### 4.1. Apparatus

IR spectra were recorded on a Nicolet 6700 Thermo-Scientific FT-IR spectrometer in KBr pellets. NMR spectra were obtained on Bruker Avance III HD (500.13 MHz for ^1^H and 125 MHz for ^13^C NMR) and Bruker Avance III (850.25 MHz ^1^H Larmor frequency) spectrometers. Commercially available broadband and solid-state double resonance probes supporting zirconia rotors with 2.5 mm outer diameter were use. Standard CP-MAS pulse sequences as well as modified versions of the Bruker pulse programs were used. To determine the quaternary carbon atoms, the cross-polarization experiments were run at different contact times and with the CPPI technique. ^13^C spectra are referenced to a glycine or alanine samples (carbonyl atoms glycine = 176.1 ppm and alanine = 177.8 ppm) as external standards. Complete signal assignments of the ^1^H NMR spectra in solution are given in the main text and in Table 1. NMR spectra in solution were obtained in DMSO-*d*_6_ solvent at concentration of 0.05 M and 298 K. Table 2 and Table 3 present the assignments of the ^13^C CP-MAS NMR data. The chemical shifts are given in parts per million (δ) in DMSO-*d*_6_ relative to the residual solvent peak [42] NMR data in solution are listed in this section to illustrate the incompleteness of the observed ^13^C-NMR spectra and for the sake of comparison with other published data. Multiplicities for proton spectra are indicated by s, singlet; br. s, broad singlet; d, doublet; t, triplet; q, quartet; m, multiplet. Coupling constants (J) are reported in Hz. Temperature-dependent ^1^H-NMR spectra were obtained for selected compounds in the temperature range of 20–80 °C and are depicted in Supplementary Materials. Melting points (m.p., °C) were taken on a microhot stage apparatus Boetius PHMK 05 and are uncorrected. BaMnO_4_ was synthesized according to the work of Kótai et al. [33].

### 4.2. Synthesis

All chemicals were of AR or synthetic grade, available from various commercial sources, and were used without further purification. Toluene, ethyl acetate, dichloromethane, acetonitrile, and light petroleum (b.p. 40–60 °C) were distilled before use. Thin-layer chromatography (TLC) was performed on Merck 1.05554 silica gel 60F254 aluminum plates, spots were visualized under UV irradiation. Column chromatography was carried out on Macherey-Nagel Kieselgel 60 (0.063–0.200 mm).

The synthesis of the alcohols **1a**–**c** was performed following several published procedures [32,37,43] and optimizing the reaction conditions depending on the solubility of the starting compounds. In general, to a mixture of Cu(CH_3_COO)_2_·H_2_O (39.93 g, 199.65 g/mol, 0.2 mol) in 200 mL 25% ammonia and dihydroxyacetone (dimer) (9.008 g, 0.05 mol, 180.16 g/mol) in 10 mL water was added a solution of 0.1 mol of the corresponding benzaldehyde in 50 mL methanol. The mixture was heated at reflux for 2 h and then cooled down to yield the copper complex that was filtered off and dissolved in diluted HCl (12%, 75 mL) with gentle heating. Solid Na_2_S was added carefully while stirring for 0.5 hours and maintaining the pH < 7. The resulting black precipitate of copper sulfide was filtered, and the aqueous layer was alkalized with NaOH. Thereby, the product was formed, filtered, and washed.

*(2-Phenyl-1H-imidazol-4(5)-yl)methanol* (**1a**), Yield 5.171 g (29.7%); brownish polycrystalline solid m.p. 127–129 °C, consistent with the literature data [37]. IR (KBr): ν (cm^−1^) 3153 (NH), 3041 (OH), 2926–2863 (CH_2_), 1578–1538 (C=N), 1484–1466 (C=N, C=C); ^1^H-NMR (DMSO-*d*_6_) *δ* (ppm) 4.43 (s, 2H, CH_2_OH), 4,97 (br. s, 1H, OH), 6.99 (s, 1H, H-4 or H-5), 7.31 (dddd, 1H, PhH, *J* = 1.2, 1.2, 6.8, 8.1 Hz,), 7.39–7.44 (m, 2H, PhH), 7.90–7.94 (m, 2H, PhH). 12.38 (br. s, 1H, H-1). ^13^C NMR (DMSO-*d*_6_) *δ* (ppm) 125.57 (2C, *C*HPh); 126.41 142.08 (1C, *C*imi); 127.45 142.08 (1C, *C*Himi); 128.91 (2C, *C*HPh); 129.28 (1C, *C*HPh); 142.08 (1C, N*C*Nimi); 185.07 (1C, *C*HO).

*(2-(4-Methoxyphenyl)-1H-imidazol-4(5)-yl)methanol* (**1b**), Yield 3.559 g (34.9%); yellowish polycrystalline powder. After recrystallization from methanol/ethyl acetate the yield was 1.323 g (13%) as needle shaped crystals; m.p. 159–161 °C; IR (KBr): ν (cm^−1^) 3118 (NH), 3044 (OH), 2975–2913 (CH_2_), 2844 (CH_3_), 1620–1577 (C=N), 1506–1452 (C=N, C=C); ^1^H NMR (DMSO-*d*_6_) *δ* (ppm) 3.78 (s, 3H, OCH_3_); 4.41 (s, 2H, OCH_2_); 4.93 (br. s, 1H, OH); 6.92 (br. s, 1H, H-imi); 6.98 (d, 2H, H-Ph, *J* = 8.9 Hz); 7.85 (d, 2H, H-Ph, *J* = 8.9 Hz); 12.24 (br s., 1H, NH); ^13^C NMR (DMSO-*d*_6_) *δ* (ppm) 55.16 (1C, OCH_3_); 56.06 (1C, OCH2); 114.05 (2C, CH-Ph); 123.74 (1C, C-Ph); 126.14 (2C, CH-Ph); 145.23 (1C, C-imi); 159.04 (1C, C-Ph).

*4-(4-(hydroxymethyl)-1H-imidazol-2-yl)benzonitrile* (**1c**), Yield 0.803 g (40%), yellowish powder m.p. 183–185 °C; IR (KBr): ν (cm^−1^) 3091 (NH), 3044 (OH), 2921–2865 (CH_2_), 2228 (CN), 1612–1576 (C=N), 1534–1492 (C=N, C=C); ^1^H NMR (DMSO-*d*_6_) δ (ppm) 4.43 (br. s, 2H, OCH_2_); 4.48 (br. s, 2H, OCH_2_*); 4.94 (br. s, 1H, OH); 5.20 (br. s, 1H, OH*); 6.97 (br. s, 1H, Imi-H); 7.19 (br. s, 1H, Imi-H*); 7.88 (d, 4H, H-Ph, H-Ph*, J = 8.4 Hz); 8.09 (br.s, 4H, H-Ph, H-Ph*); 12.71 (br. s, 1H, NH); 12.85 (br, 1H, NH*); ^13^C NMR (DMSO-*d*_6_) *δ* (ppm) 57.62 (1C, OCH2); 109.70 (1C, PhC); 118.92 (1C, CN); 125.03 (2C, PhCH); 132.80 (2C, PhCH); 134.82 (1C, PhC).

*2-Phenyl-1H-imidazole-4(5)-carbaldehyde* (**2a**), Solution of **1a** (1.472 g, 174.2 g/mol; 8.5 mmol) in 65% nitric acid (4 mL) was heated with stirring at 100 °C for 1 hour (the reaction was followed by TLC). Reaction mixture was diluted with water (20 mL) and the pH was adjusted to 8 with 10% Na_2_CO_3_. After cooling, the formed precipitate was filtered and washed with water. Additional amount of the aldehyde was acquired from the aqueous layer after saturation with salt, extraction with ethyl acetate (3 × 20 mL), and evaporation of the organic layers after being dried with Na_2_SO_4_, Yield 0.507 g (35%). M. p. 158-160 °C; IR (KBr): ν (cm^−1^) 3143–3087 (NH), 1656 (C=O), 1528–1548 (C=N), 1485–1456 (C=N, C=C); ^1^H NMR (DMSO- *d*_6_) δ (ppm) 7.41–7.47 (m, 1H, H-Ph); 7.49–7,53 (m, 2H, H-Ph); 7.91–8.21 (br, 3H, 2xH-Ph, H-Imidazole); 9.79 (br, 1H, CHO); 13.36 (br, 1H, NH): 13.58 (br, 1H, NH); ^13^C NMR (DMSO- *d*_6_) δ (ppm) 126.50 (2C, C-Ph); 127.30 (1C, C-Ph); 128.38 (1C, C-Ph); 129.84 (2C, C-Ph); 130.45 (1C, C-imidazole); 130.77 (1C, C-imidazole); 143.00 (1C, C-imidazole); 185.97 (1C, CHO).

*2-(4-methoxyphenyl)-1H-imidazole-4(5)-carbaldehyde* (**2b**), The alcohol **1b** (2.850 g, 204.23 g/mol, 0.014 mol) was dissolved in toluene/dimethyl formamide (50 mL/10 mL) and heated to 100 °C before adding the BaMnO_4_ (7.17 g, 256.26 g/mol, 0.028 mol). The mixture was stirred at 100 °C for 2 h and then filtered through layer of silica gel and washed with ethyl acetate. The solvent was evaporated that yielded 2.511 g (89%) of dark brown oily product, which was purified by column chromatography (light petroleum/ethyl acetate = 1/1) on silica gel (MN 60). The solvent was removed under reduced pressure and the product solidifies yielding 0.840 g (29%); yellow crystals. M.p. 164–166 °C; IR (KBr): ν (cm^−1^) 3139 (NH), 2951–2840 (CH_2_, CH_3_), 1672–1646–1618 (C=N), 1500–438 (C=N, C=C); ^1^H NMR (DMSO- *d*_6_) δ (ppm) 3.81 (s, 6H, OCH3, OCH3*); 7.06 (d, 4H, H-Ph, H-Ph*, *J* = 8.9 Hz); 7.95 (br.s, 3H, H-Ph, H-imi); 8.10 (br. s, 3H, H-Ph*, H-imi*); 9.68 (br. s, 1H, CHO); 9.74 (br. s, 1H, CHO*); 13.19 (br, 1H, NH); 13.39 (br, 1H, NH*); ^13^C NMR (DMSO- *d*_6_) δ (ppm) 55.34 (2C, OCH3, OCH3*); 114.35 (4C, CH-Ph, CH-Ph*); 122.18 (2C, C-Ph, C-Ph*); 127.14 (2C, CH-Ph); 128.17 (2C, CH-Ph*); 142.18 (2C, C-imi, C-imi*); 160.04 (2C, C-Ph, C-Ph*); 184.92 (2C, CHO, CHO*).

*4-(4-formyl-1H-imidazol-2-yl)benzonitrile* (**2c**), The synthetic procedure for **2b** was followed using 2 mmol of **1c**, Yield 0.190 g (48%); white crystals, sublimation around 170 °C. IR (KBr): ν (cm^−1^) 3142 (NH), 2971–2876 (CH_2_), 2228 (CN), 1668–1610 (C=N), 1493–1434 (C=N, C=C); ^1^H NMR (DMSO- *d*_6_) δ (ppm) 7.97 (d, 2H, *H*-Ph, *J* = 8.5 Hz); 8.21 (br. s, 3H, 2×*H*-Ph, H-imi); 9.81 (s, 1H, C*H*O); 13.73 (br. s, 1H, N*H*); ^13^C NMR (DMSO- *d*_6_) δ (ppm) 111.52 (1C, *C*Ph); 118.60 (1C, *C*N); 126.36 (2C, *C*HPh); 132.98 (2C, *C*HPh); 133.47 (1C, CPh).

*2-(4-hydroxyphenyl)-1H-imidazole-4(5)-carbaldehyde* (**2d**), The hydroxyphenyl-substituted aldehyde was obtained from **2b** (0.708 g, 3.5 mmol) by heating it for 15 h at 100 °C in 48 % HBr (10 mL) and following the reaction with TLC. After cooling down, the reaction mixture was treated with 10% Na_2_CO_3_ (3 mL), and additional solid Na_2_CO_3_ until pH = 9, to form crystalline product. The crude product was filtered and purified by column chromatography on silica gel (MN 60) and eluent light petroleum/ethyl acetate 1/1, Yield 0.268 g (40.7%); yellow powder. M.p. 269–270 °C; IR (KBr): ν (cm^−1^) 3244 (OH), 3054 (NH), 2924–2857 (CH_2_), 1661–1612–1586 (C=N), 1488–1459–1418 (C=N, C=C); ^1^H NMR (DMSO- *d*_6_) δ (ppm) 6.85 (d, 4H, H-Ph, H-Ph*, *J* = 8.7 Hz); 7.90 (br.s, 4H, H-Ph, H-Ph*); 8.01 (br. s, 2H, H-imi, H-imi*); 9.64 (br. s, 1H, CHO); 9.70 (br. s, 1H, CHO*); 9,92 (br. s, 2H, OH, OH*); 12.11 (br, 1H, NH); 13.14 (br, 1H, NH*); ^13^C NMR (DMSO- *d*_6_) δ (ppm) 115.62 (4C, CH-Ph, CH-Ph*); 120.48 (2C, C-Ph, C-Ph*); 127.76 (4C, CH-Ph, CH-Ph*); 131.63 (2C, CH-Ph*); 131.78 (2C, C-imi, C-imi*); 158.88 (1C, C-Ph, C-Ph*); 191.35 (2C, CHO, CHO*). Calcd. for (C_10_H_8_N_2_O_2_): C 63.83%; H 4.29%; N 14.89%; found: C 64.3%; H 4.7%; N 14.4%.

### 4.3. Computational Methods

The quantum chemical calculations were performed with the DFT methods implemented in the Gaussian 09 suite of programs [44]. Based on our previous experience, the hybrid B3LYP functional, which combines the Becke exchange [45] and LYP correlation functionals [46,47], was selected and applied for all geometry optimizations in combination with the split-valence basis set of double-zeta quality including diffuse functions 6-31G(d,p) [48,49]. Vibrational frequencies and intensities were computed at the same level of theory for all optimized structures and confirmed the attainment of local minima. The ^13^C-NMR shielding constants were calculated using the gauge-including atomic orbitals, GIAO-DFT, method [50], and the 6-31G(d,p) basis set. The calculated isotropic NMR shielding constants, σ_i_, were converted to ^13^C chemical shifts using the equation: δ_i_ = 191.818 − σ_i_, where the value of 191.818 is the shielding constants of TMS calculated with the same methods and used as reference.

## Figures and Tables

**Table 1:** Assignments of the signals from the ^1^H NMR spectra of compounds **1a**–**c** and **2a**–**d**, all obtained in DMSO-*d*_6_ at 298 K and concentration 0.05 M. Atoms numbering is shown in Figure 1.

	Number H-Atom	H5	H6	H8	H9	H10 (*-OMe)	OH	NH
Compound								
**1a**		6.99 (s)	4.43 (s)	7.39–7.44 (m, 2H)	7.90–7.94 (m, 2H)	7.31 (dddd)	4.97 (bs)	12.38 (bs)
**1b**		6.92 (bs)	4.41 (s)	7.85 (d, 2H)	6.98 (d, 2H)	* 3.78 (s)	4.93 (bs)	12.24 (bs)
**1c**		7.19 (bs) 6.97 (bs)	4.43 (bs) 4.48 (bs)	8.09 (bs, 4H)	7.88 (d, 4H)	─	4.94 (bs) 5.20 (bs)	12.71 (bs) 12.85 (bs)
**2a**		7.90–8.21 (bs)	9.79 (bs)	7.90–8.21 (bs)	7.49–7.53 (m)	7.41–7.47 (m)	─	13.36 13.58
**2b**		7.95 (bs) 8.10 (bs)	9.68 (bs) 9.74 (bs)	7.95 (bs) 8.10 (bs)	7.06 (d, 4H)	* 3.81 (s)	─	13.19 (bs) 13.39 (bs)
**2c**		8.21 (bs)	9.81 (s)	8.21 (bs)	7.97 (d, 2H)	─	─	13.73 (bs)
**2d**		8.01 (bs)	9.64 (bs) 9.70 (bs)	7.90 (bs, 4H)	6.85 (d, 4H)	─	9.92 (bs)	12.11 (bs) 13.14 (bs)

S—singlet; bs—broad singlet; m—multiplet; d—doublet. *—for **1b** and **2b** the values correspond to methyl protons instead of H10.

**Table 2:** Experimental (CP-MAS in bold) and calculated (GIAO-B3LYP-6-31G(d,p)) ^13^C NMR shifts of compounds **1a**–**d** (the shifts for quaternary carbon atoms are underlined). Calculated data are given for the two most stable forms (form **I** and form **II**), shown in Figure 1 and Figure 7. Compound **1d** is studied only theoretically. For compound **1b**, distinct signals for tautomers **I** and **II** could be assigned from the cross-polarization/polarization-inversion (CPPI) and ^1^H-^13^C HETCOR MAS NMR spectra.

	Number C-Atom	C2	C4	C5	C6	C7	C8	C9	C10	CN or Me
Compound										
**1a–I**		139.20	141.11	105.47	58.72	125.38	122.19 115.83	124.09 127.73	122.28	─
**1a–II**		139.61	122.08	124.99	58.31	126.35	116.07 122.19	122.45 123.79	121.58	─
**1a** **CP-MAS**		**149.4**	**141.6**	**115.8**	**54.8**	**128.9**	**127.9**	**128.9**	**124.6**	
**1b–I**		139.17	140.68	104.75	58.74	118.74	124.01 117.22	114.08 104.04	153.17	52.52
**1b–II**		139.77	122.03	124.24	58.33	119.59	117.73 123.97	112.14 105.56	152.65	52.43
**1b** **CP-MAS**		**146.6** **_(I)_** **146.9** **_(II)_**	**139.3_(I)_** **109.6_(II)_**	**108.7_(I)_** **130.0_(II)_**	**54.2**	**123.3**	**127.0** **128.2** **129.4**	**117.2** **116.4** **130.0**	**159.9**	**55.8**
**1c–I**		137.34	141.36	109.43	62.02	128.93	122.13 115.35	128.80 127.97	108.83	109.84
**1c–II**		137.72	122.31	127.08	58.09	129.20	115.89 122.02	127.94 128.75	108.83	109.92
**1c** **CP-MAS**		**145.7**	**142.4**	**127.3**	**55.3**	**132.8**	**127.3**	**132.8**	**110.7**	**120.3**
**1d–I**		139.01	140.70	104.76	58.73	118.56	117.05 124.85	107.79 111.62	150.24	─
**1d–II**		139.62	121.97	124.31	58.30	119.52	118.33 123.91	109.53 109.44	149.75	─

**Table 3:** Experimental (CP-MAS in bold) and calculated (GIAO-B3LYP-6-31G(d,p)) ^13^C NMR shifts of compounds **2a–d** (the shifts for quaternary carbon atoms are underlined). Calculated data are given for the two most stable forms (form **I_R** and form **II** as depicted in Figure 7), shown in Figure 1 and Figure 7. For some C-atoms of compounds **2c** and **2d**, distinct signals for tautomers **I** and **II** could be assigned from the CPPI and/or ^1^H-^13^C HETCOR MAS NMR spectra and are indicated as subscripts.

	Number C-Atom	C2	C4	C5	C6	C7	C8	C9	C10	CN or Me
Compound										
**2a–I_R**		140.24	140.03	114.51	178.14	124.96	122.82 116.13	124.38 122.78	123.18	─
**2a–II**		143.56	133.74	129.12	168.45	124.41	117.86 124.25	123.08 124.18	124.29	─
**2a CP-MAS**		**150.0**	**129.4**	**139.7**	**183.9**	**129.4**	**126.8**	**129.4**	**129.4**	─
**2b–I_R**		140.22	139.84	114.05	178.15	118.11	124.56 117.57	114.43 104.09	153.82	52.62
**2b–II**		143.55	133.93	128.87	167.97	117.46	119.69 125.96	104.58 114.15	154.71	52.53
**2b** **CP-MAS**		**149.2**	**142.3**	**139.1**	**183.6**	**121.5**	**126.6**	**111.7** **116.0**	**160.3**	**55.2**
**2c–I_R**		138.92	140.70	115.73	177.92	127.82	122.90 116.12	129.23 128.15	110.78	109.38
**2c–II**		141.86	133.61	129.79	169.24	127.39	117.84 124.19	128.25 129.04	111.80	109.37
**2c** **CP-MAS**		**146.7 ** **_(I)_** **147.0 ** **_(II)_**	**131.4**	**141.5 _(I)_** **131.2 ** **_(II)_**	**186.8**	**131.4**	**125.3** **127.0**	**131.4** **133.8**	**112.3**	**119.9**
**2d–I_R**		140.06	139.85	114.05	178.13	117.94	125.47 117.39	111.98 107.80	150.89	─
**2d–II**		143.34	133.81	128.83	168.01	117.38	119.43 126.90	108.29 111.75	151.79	─
**2d** **CP-MAS**		**152.4 _(II)_** **161.0 _(I)_**	**144.1**	**132.9 _(II)_**	**178.1**	**117.6**	**128.1** **130.0**	**1c15.7** **117.6**	**161.2**	─

## Supplementary Materials

The following are available online. 56 figures with experimental spectra.
