# Supplementary material for: 13C CPMAS NMR as a Tool for Full Structural Description of 2-Phenyl Substituted Imidazoles That Overcomes the Effects of Fast Tautomerization
Source: Molecules. 2020 Aug 19;25(17):3770. doi: 10.3390/molecules25173770 (PMC7504167; doi:10.3390/molecules25173770)
Supplement: Supplementary file 1 [file molecules-25-03770-s001.pdf]

Supplementary materials for

^{13}C CPMAS NMR as a tool for full structural description of 2-phenyl substituted imidazoles that overcomes the effects of fast tautomerization

Nikola Burdzhiev, Anife Ahmedova, Boris Borrisov and Robert Graf

Table of Contents

Spectra of (2-phenyl-1H-imidazol-4-yl)methanol (1a) 2

Spectra of (2-(4-methoxyphenyl)-1H-imidazol-4-yl)methanol (1b) 5

Spectra of 4-(4-(hydroxymethyl)-1H-imidazol-2-yl)benzonitrile (1c) 9

Spectra of 2-phenyl-1H-imidazole-4(5)-carbaldehyde (2a) 12

Spectra of 2-(4-methoxyphenyl)-1H-imidazole-4-carbaldehyde (2b) 15

Spectra of 4-(4-formyl-1H-imidazol-2-yl)benzonitrile (2c) 20

Spectra of 2-(4-hydroxyphenyl)-1H-imidazole-4-carbaldehyde (2d) 25

Spectra of 2-methyl-1H-imidazole-4-carbaldehyde 28

Spectra of (2-phenyl-1H-imidazol-4-yl)methanol (1a).

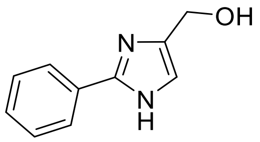

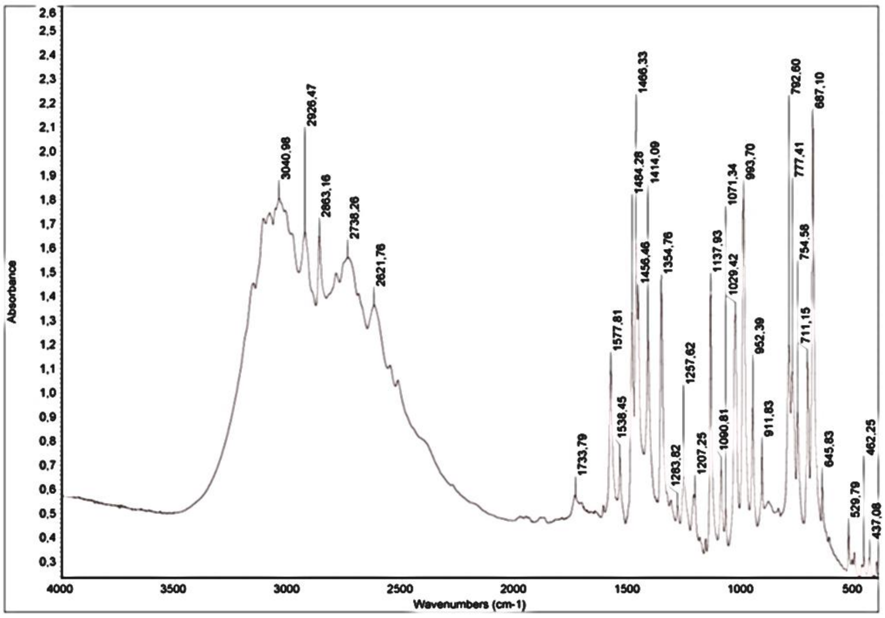

Figure 1. IR spectrum of **1a** in KBr pellets.

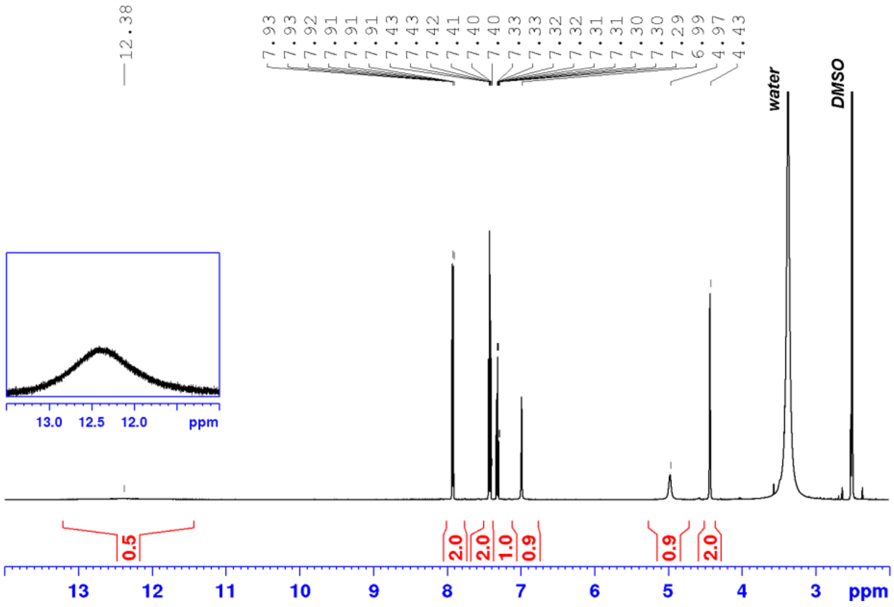

Figure 2. ^{1}H NMR (DMSO-d_6) of **1a** at 298 K.

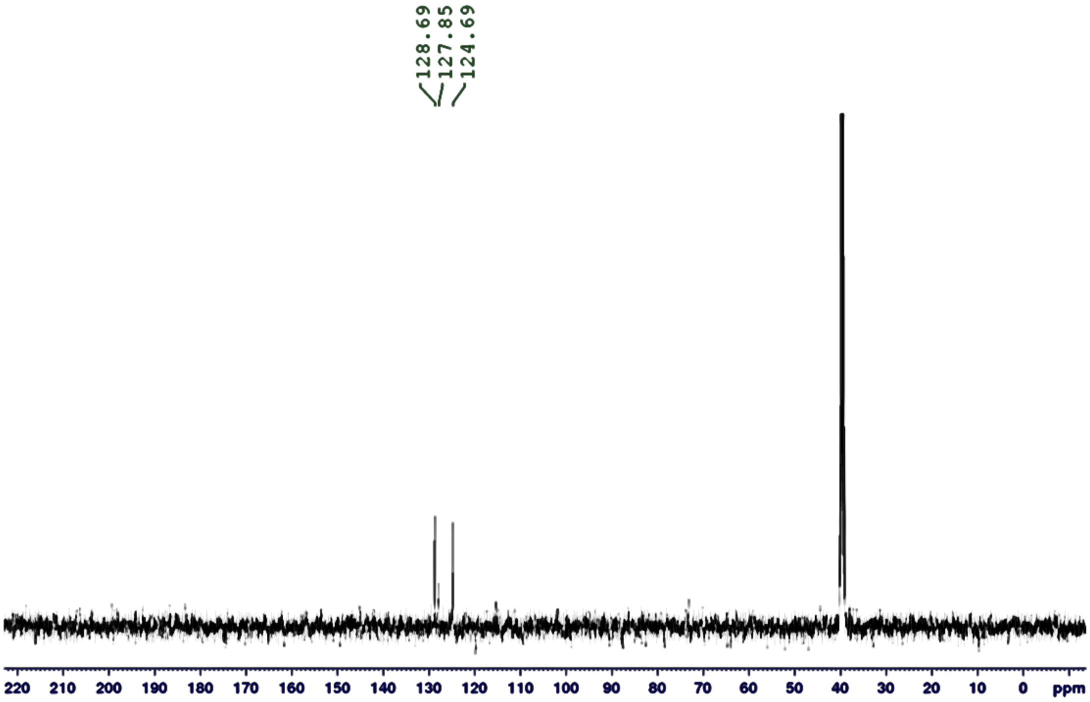

Figure 3. ^{13}C NMR (DMSO-d_6) of **1a**.

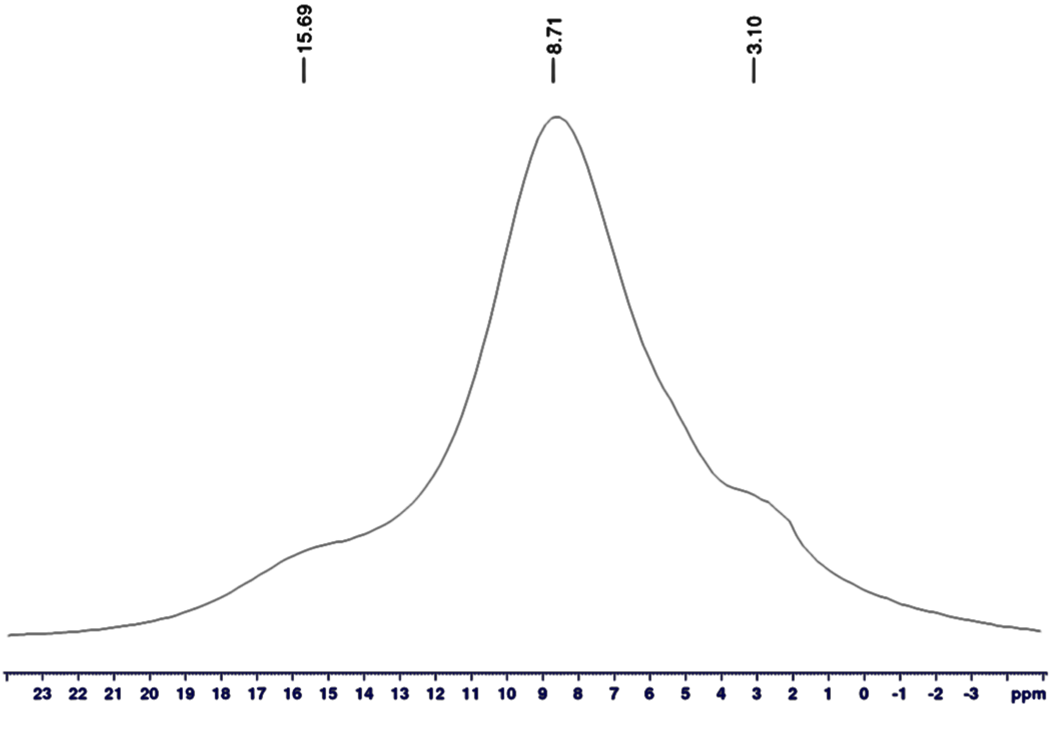

Figure 4. ^{1}H-MAS NMR (solid state 500MHz, 15kHz) of **1a**.

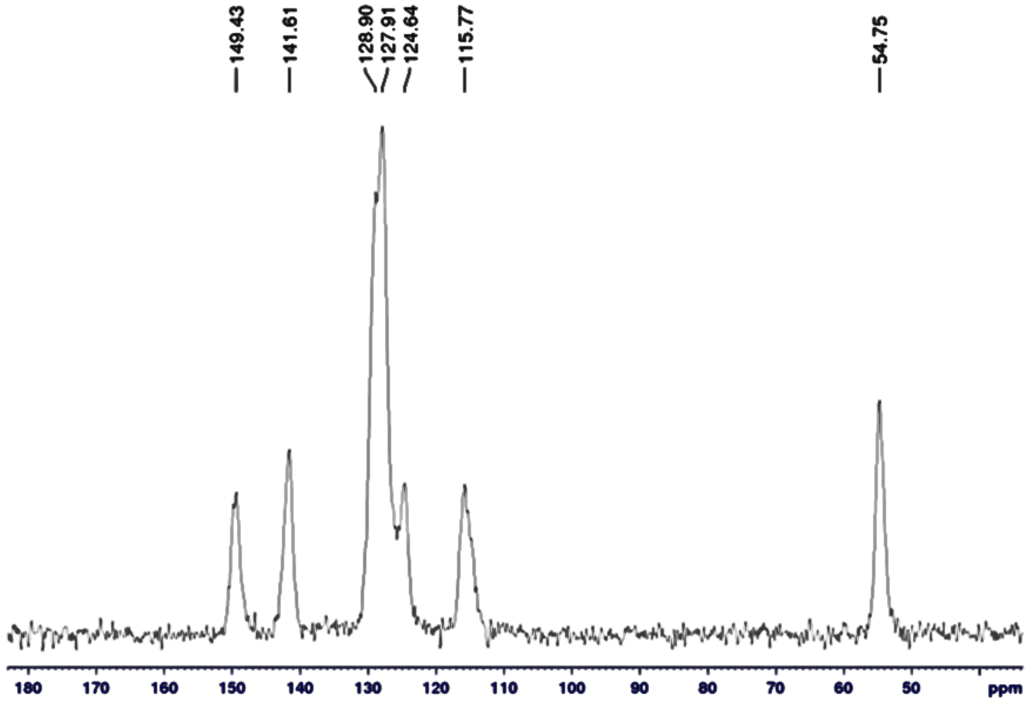

Figure 5. ^{13}C CP-MAS (solid state 125MHz, 15kHz) of **1a**.

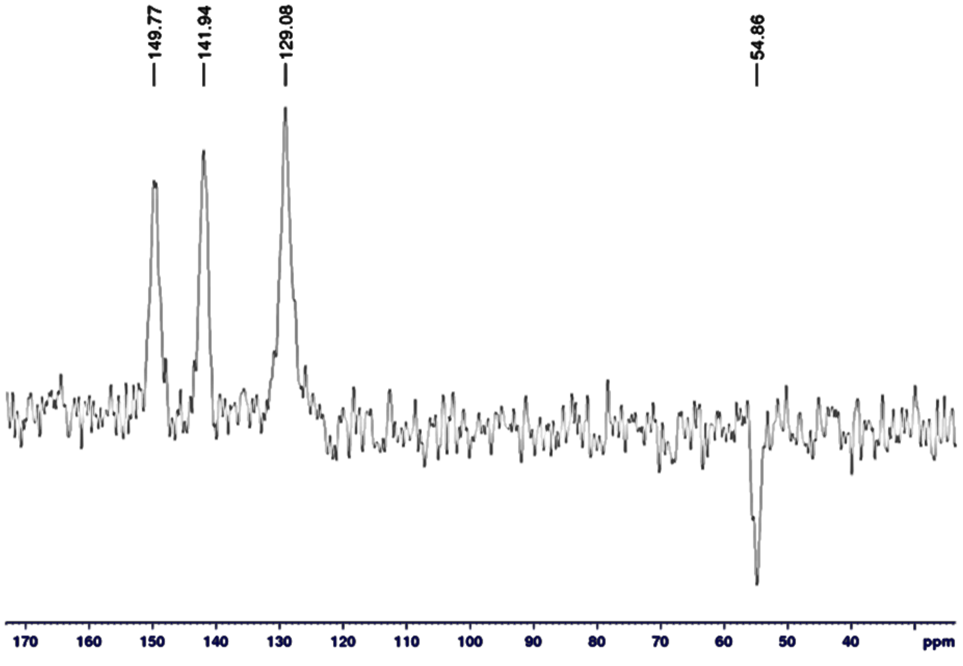

Figure 6. ^{13}C CPPI spectrum of **1a**; null signal for CH, negative signal for CH_2, and positive signal for C and CH_3.

Spectra of (2-(4-methoxyphenyl)-1H-imidazol-4-yl)methanol (1b)

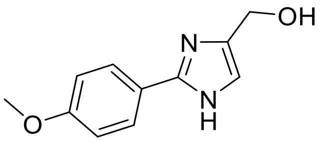

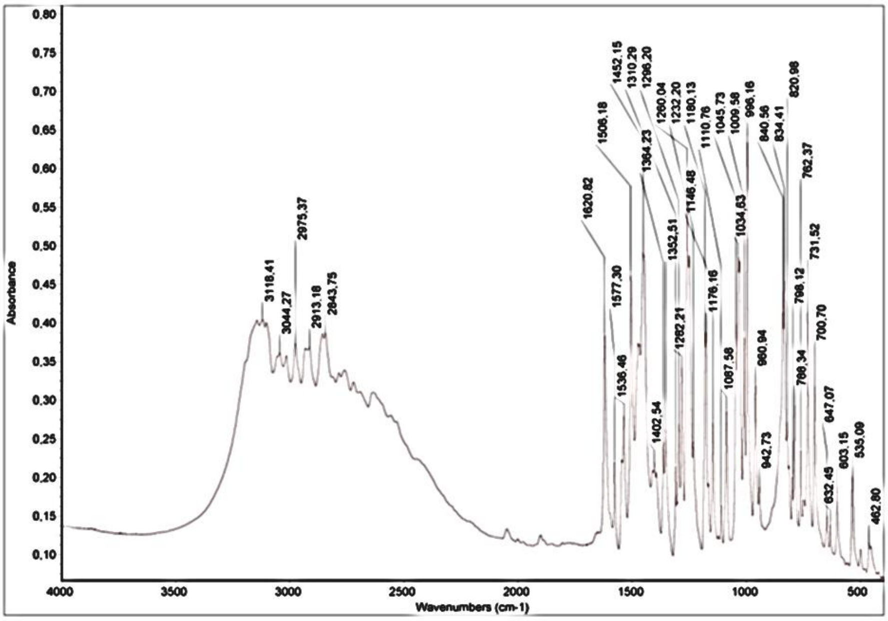

Figure 7. IR spectrum of **1b** in KBr pellets.

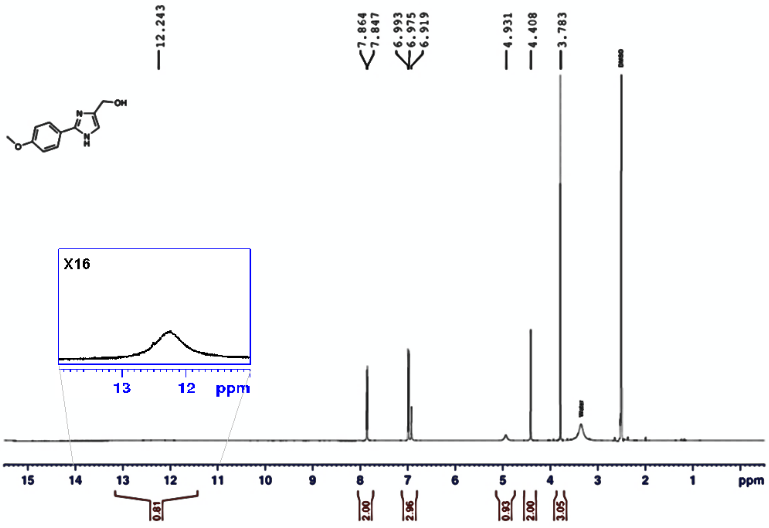

Figure 8. ^{1}H NMR (DMSO-d_6) of **1b**.

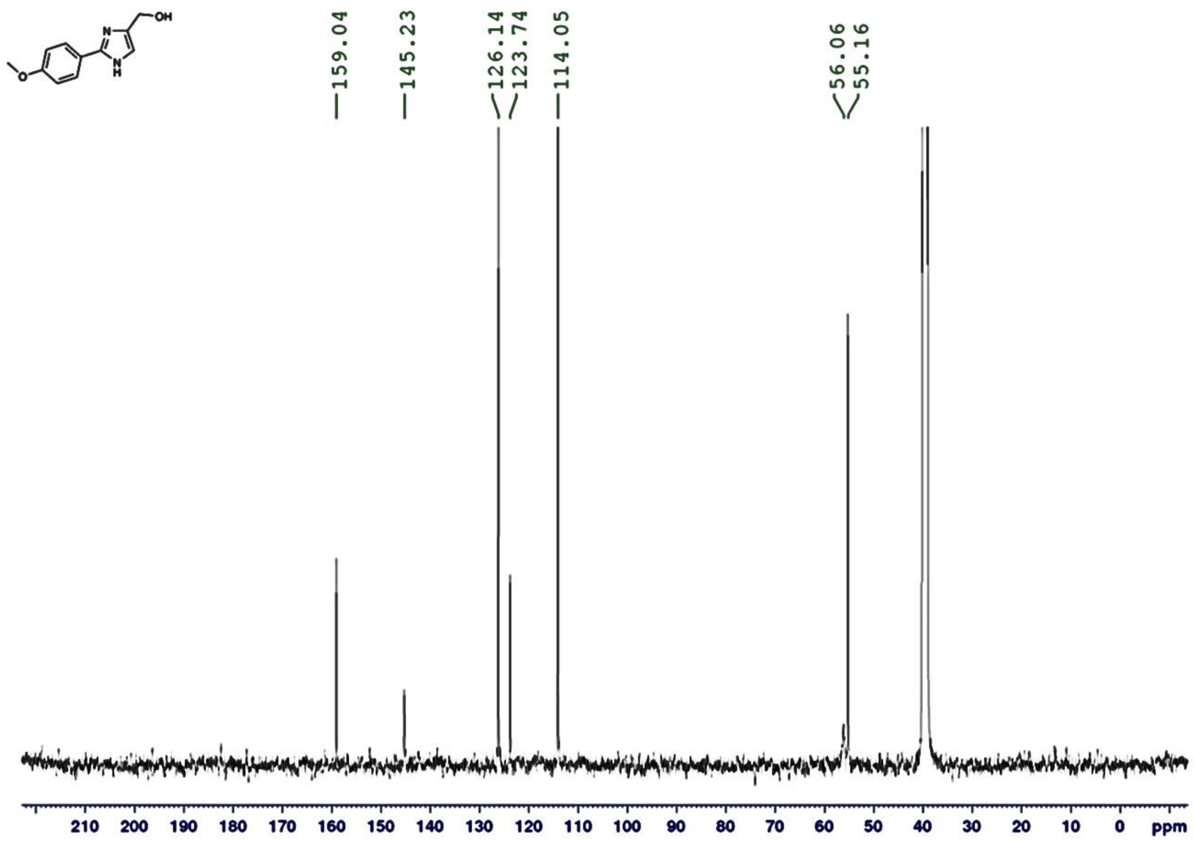

Figure 9. ^{13}C NMR (DMSO-*d*$_6$) of **1b**.

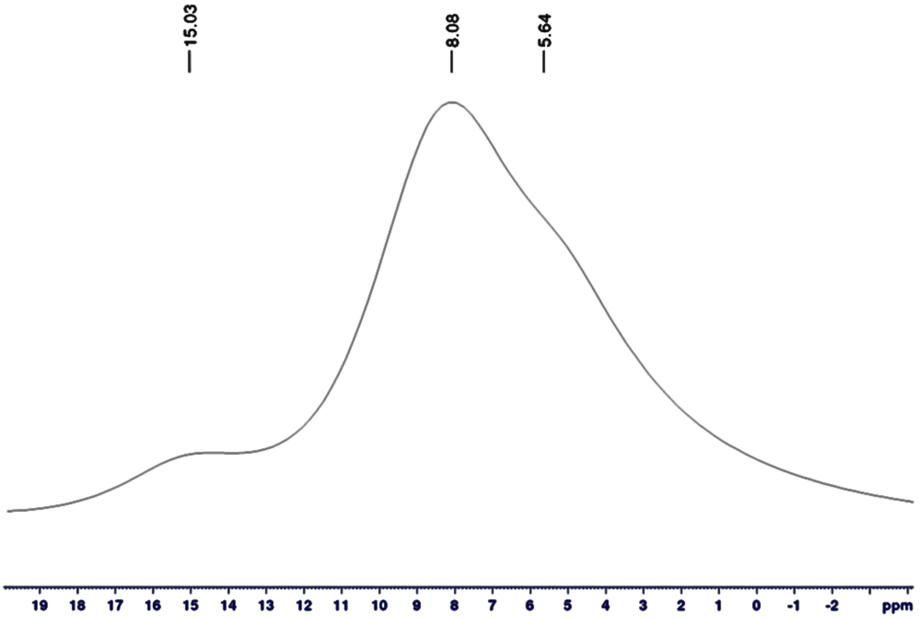

Figure 10. ^{1}H-MAS NMR (solid state 500MHz, 15kHz) of **1b**.

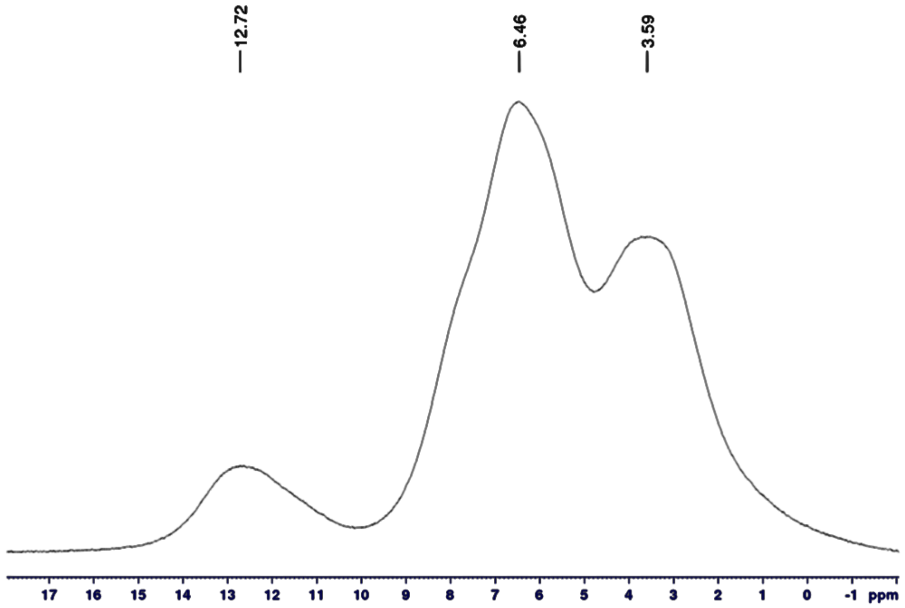

Figure 11. ^{1}H-MAS NMR (solid state 850MHz, 25kHz) of **1b**.

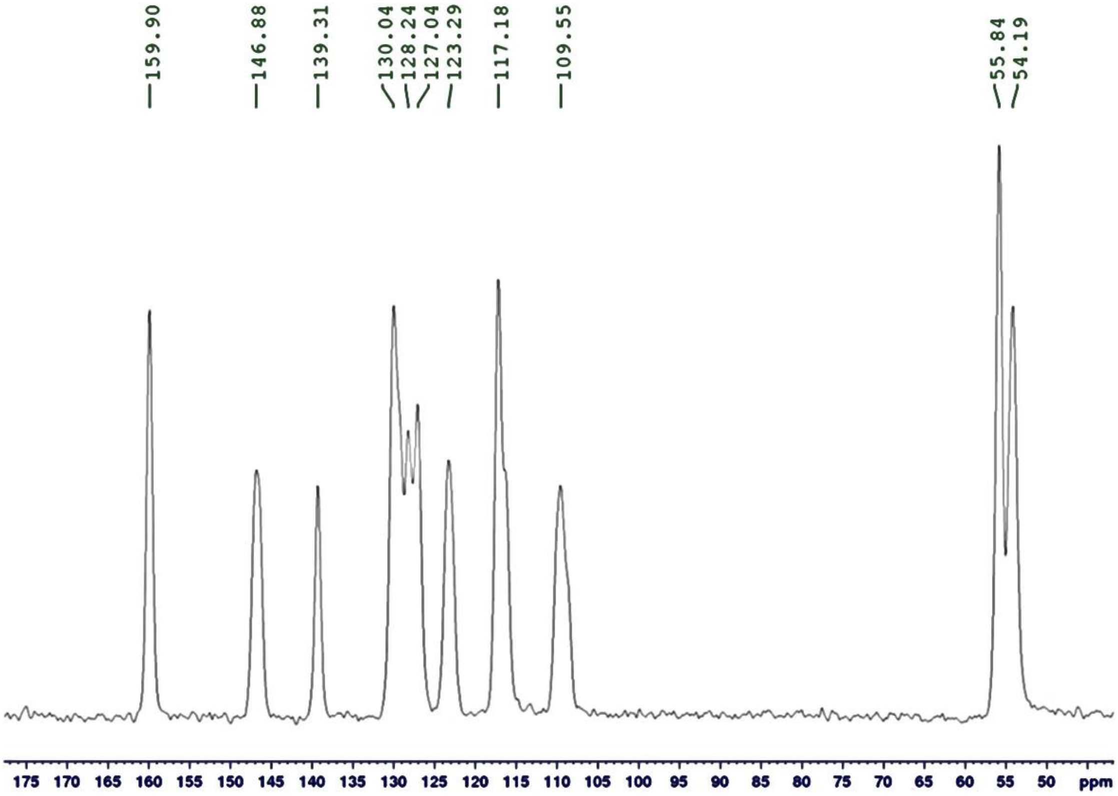

Figure 12. ^{13}C CP-MAS (solid state 125MHz, 15kHz) of **1b**; weak shoulders on the signals at 117.17 and 130.04 ppm and broadening of the signals at 109.55 and 146.88.

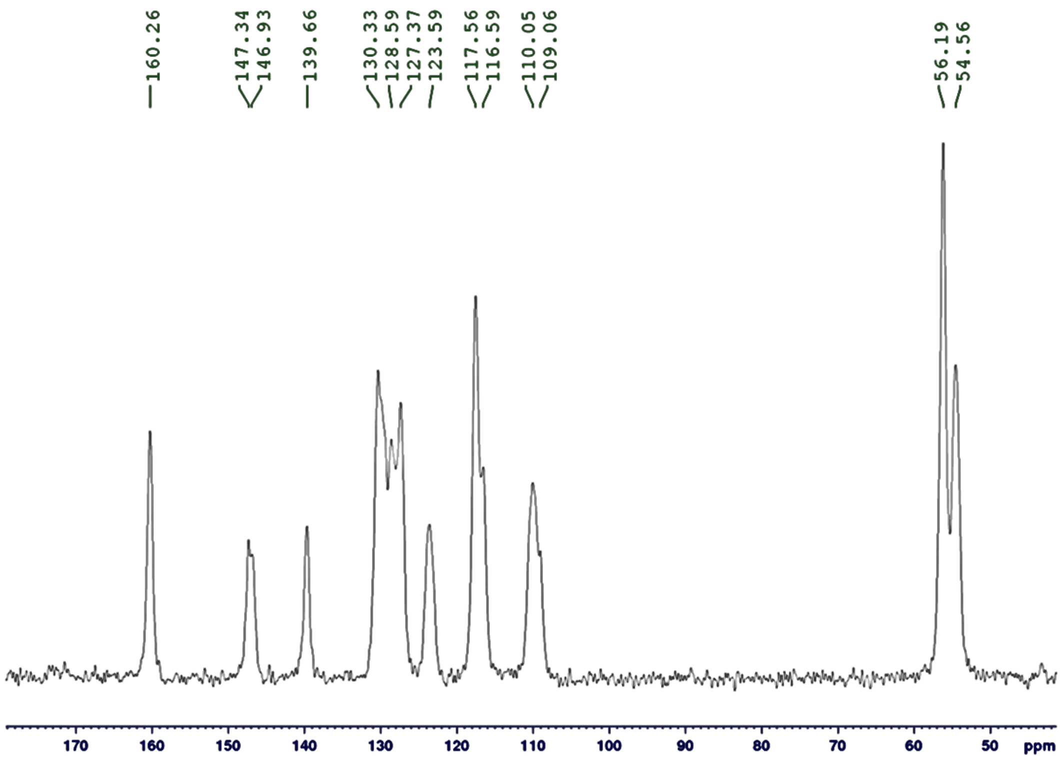

Figure 13. ^{13}C CP-MAS (solid state 212.5 MHz, 25kHz) of **1b.**

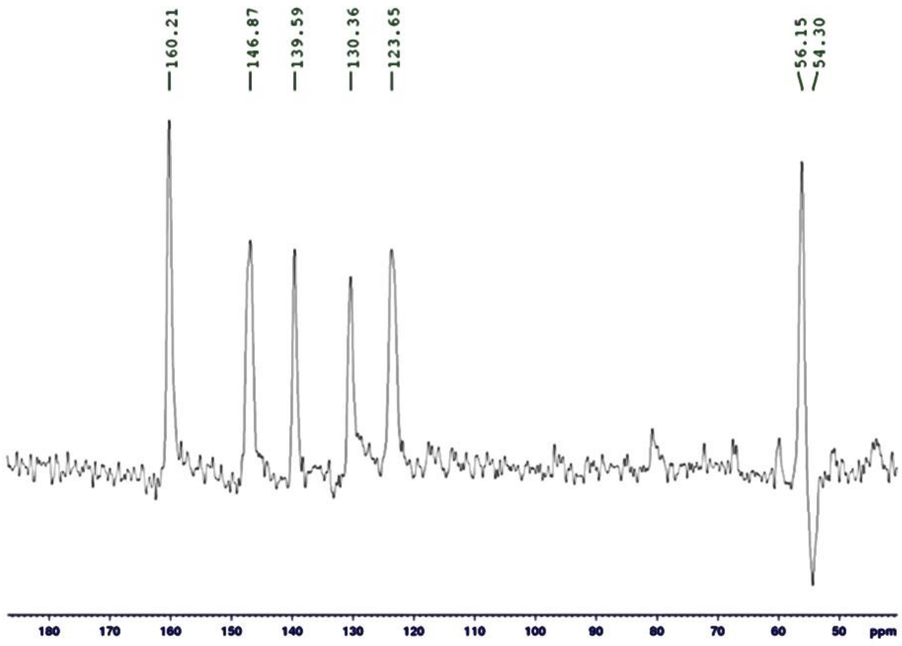

Figure 14. ^{13}C CPPI spectrum (125 MHz, 10 kHz) of **1b**; null signal for CH, negative signal for CH_2, and positive signal for C and CH_3.

Spectra of 4-(4-(hydroxymethyl)-1H-imidazol-2-yl)benzonitrile (1c)

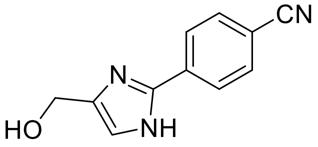

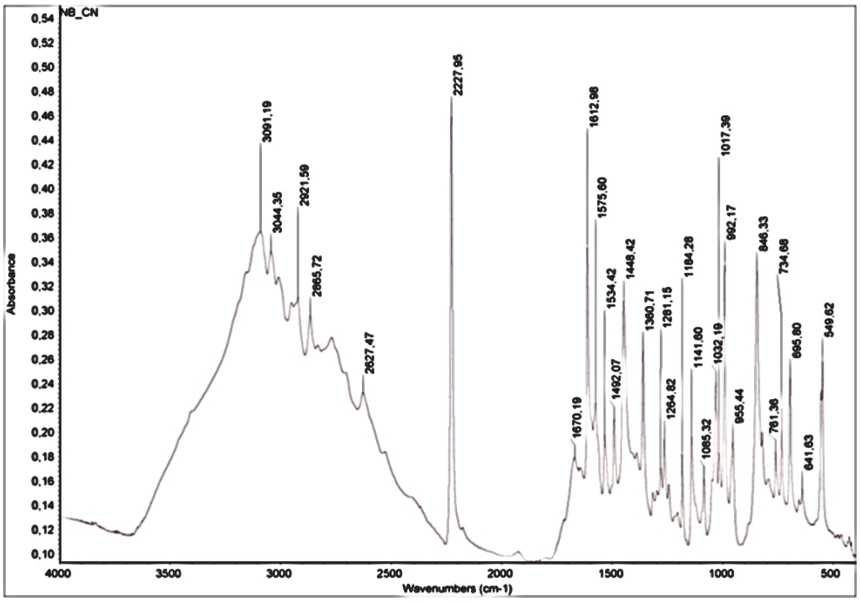

Figure 15. IR spectrum of **1c** in KBr pellets.

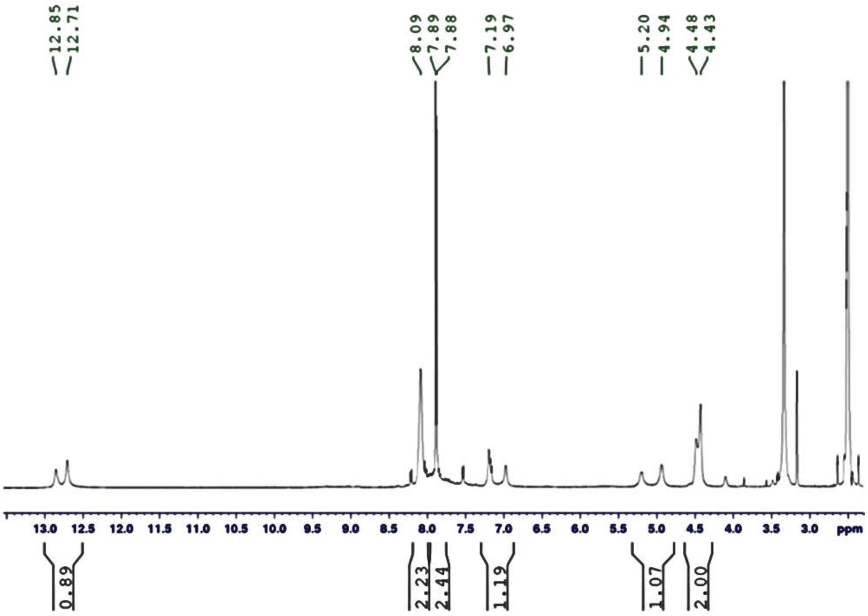

Figure 16. ^{1}H NMR (DMSO-d_6) of **1c** at 298 K.

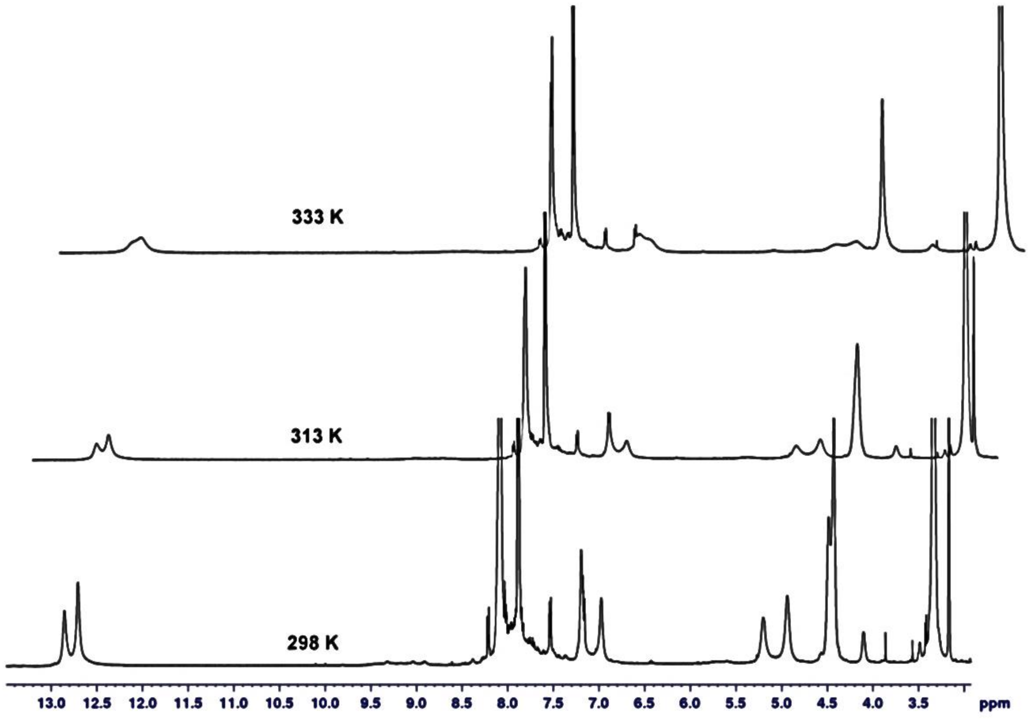

Figure 17. Temperature dependent ^{1}H NMR (DMSO-d_6) of **1c**.

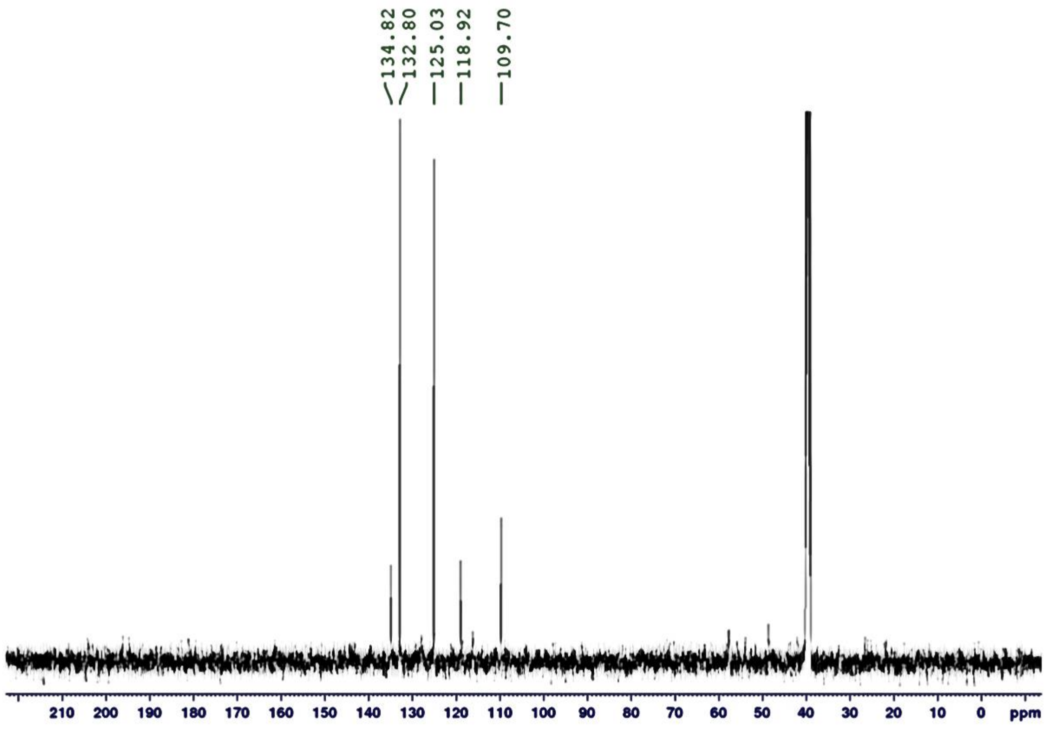

Figure 18. ^{13}C NMR (DMSO-d_6) of **1c** at 298 K.

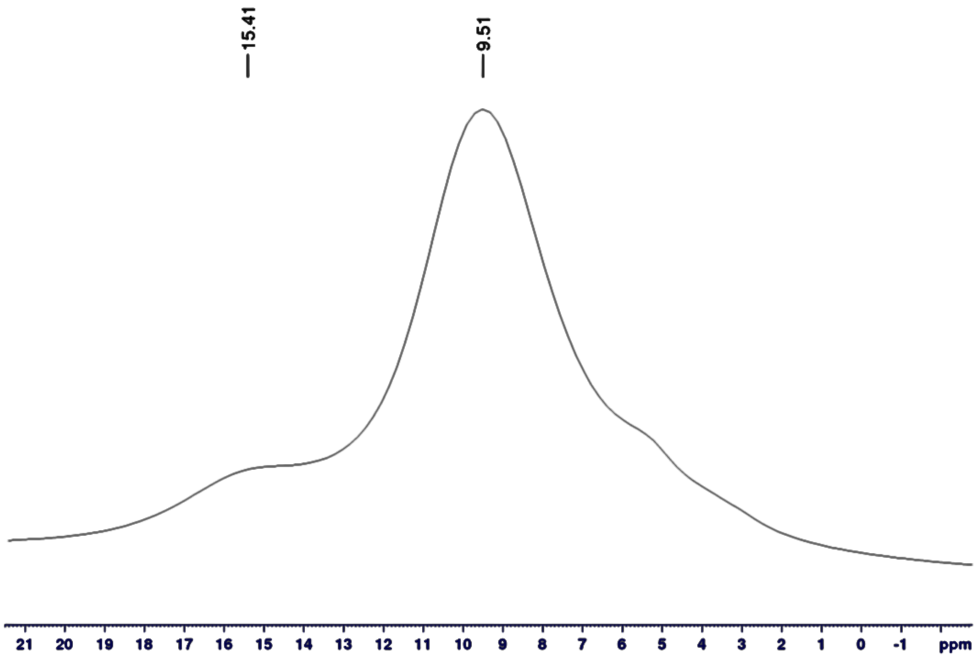

Figure 19. ^{1}H-MAS NMR (solid state 500MHz, 15kHz) of **1c**.

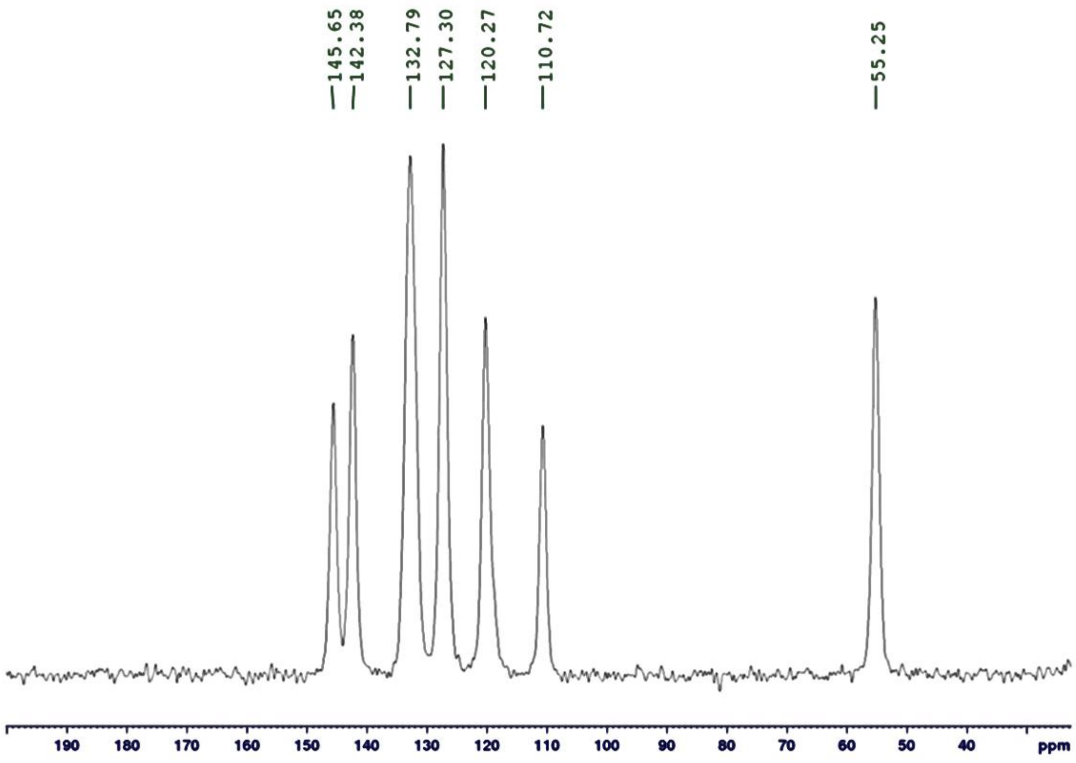

Figure 20. ^{13}C CP-MAS (solid state 125MHz, 15kHz) of **1c**.

Spectra of 2-phenyl-1H-imidazole-4(5)-carbaldehyde (2a)

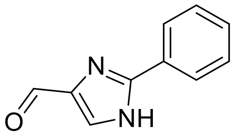

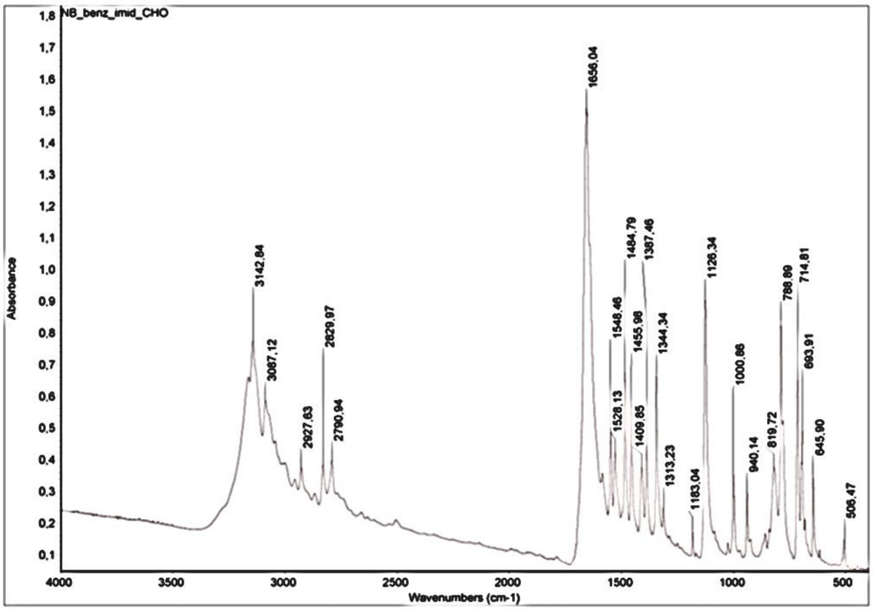

Figure 21. IR spectrum of **2a** in KBr pellets.

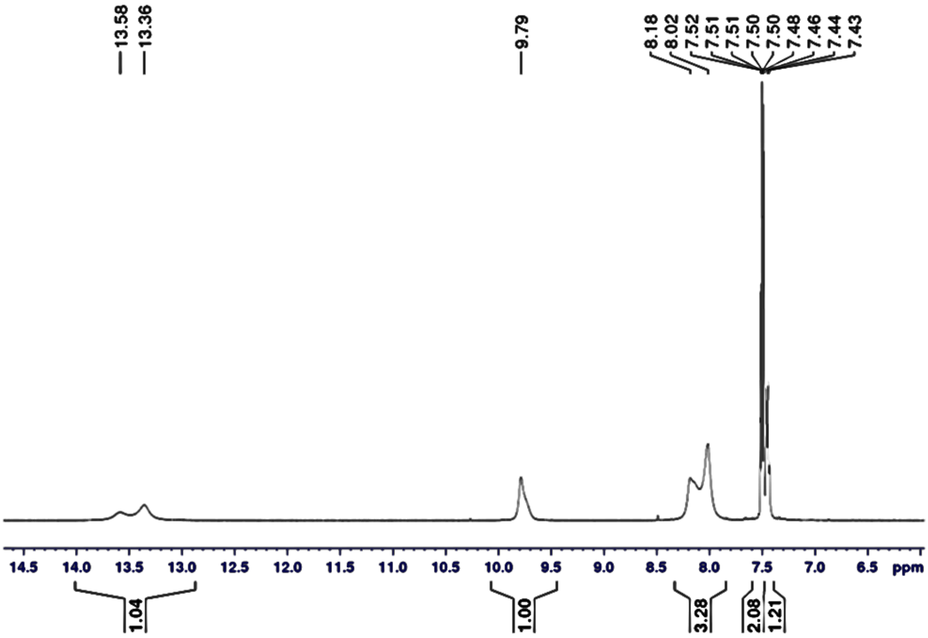

Figure 22. ^{1}H NMR (DMSO-d_6) of **2a** at 298 K.

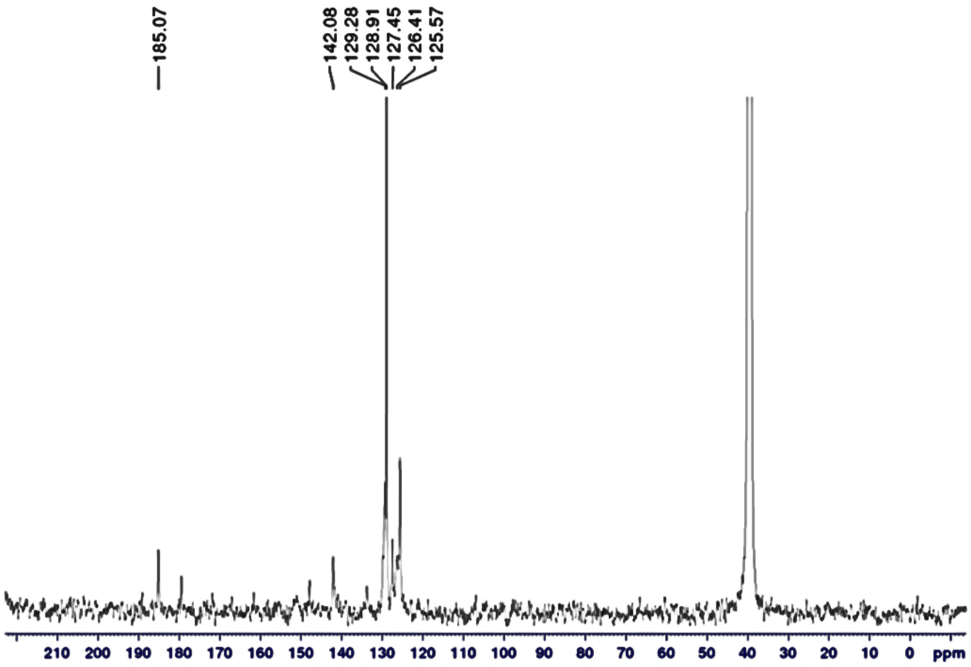

Figure 23. ^{13}C NMR (DMSO-d_6) of **2a** at 298 K (125MHz; NS 512).

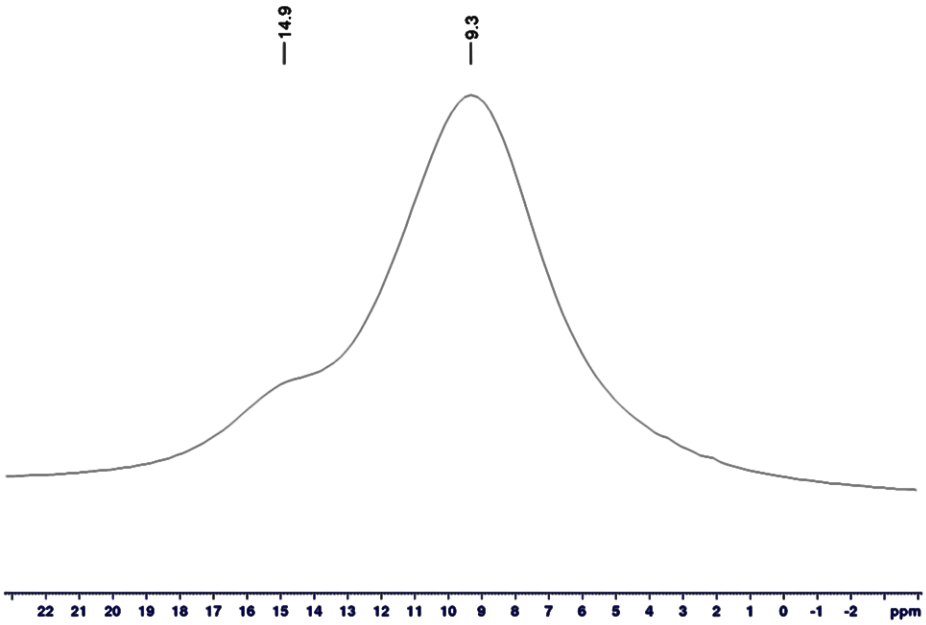

Figure 24. ^{1}H MAS NMR (solid state 500MHz, 15kHz) of **2a.**

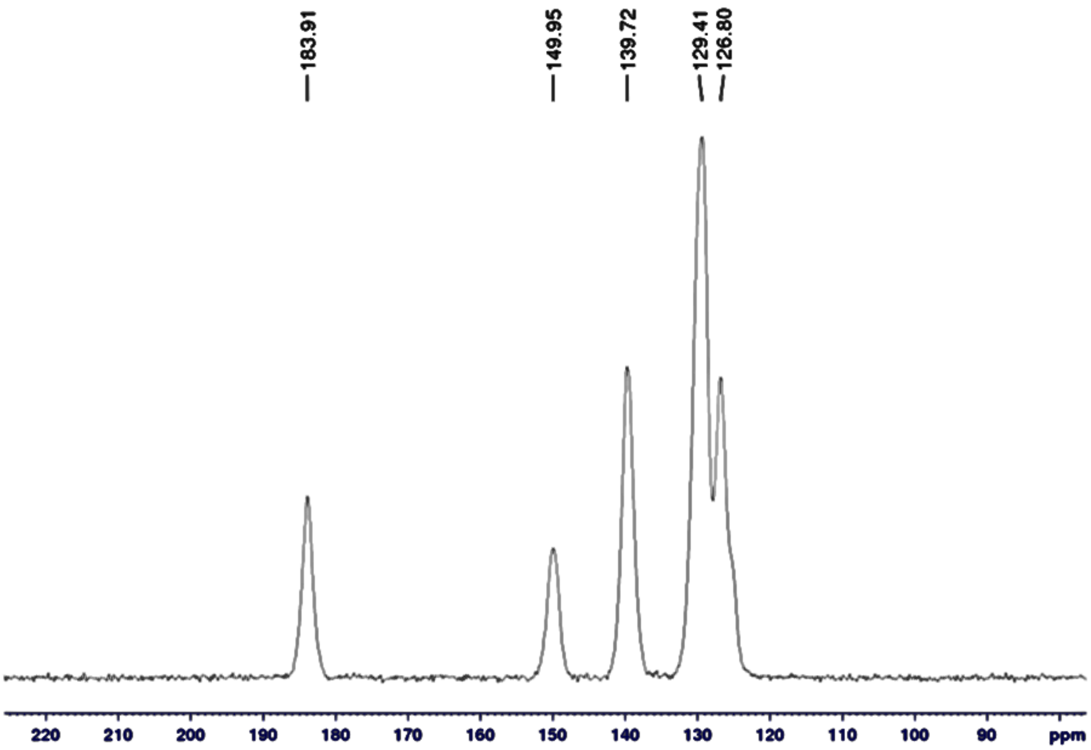

Figure 25. ^{13}C CP-MAS NMR (solid state 500MHz, 15kHz) of **2a.**

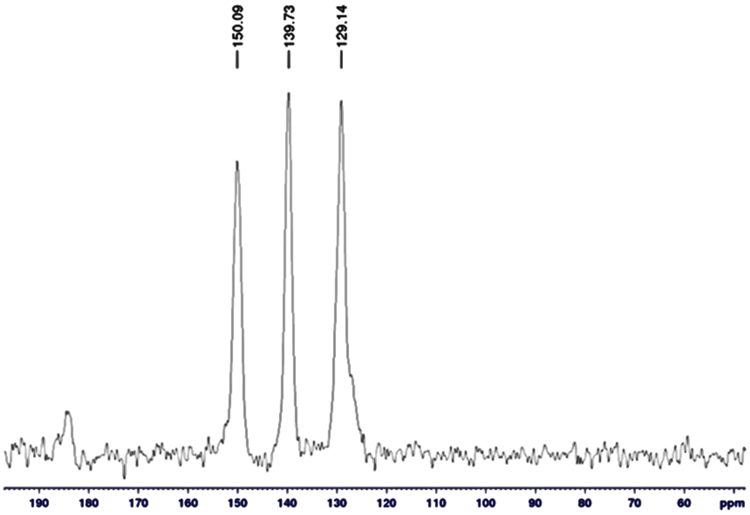

Figure 26. ^{13}C CPPI spectrum (125 MHz, 10 kHz) of **2a**; null signal for CH, negative signal for CH_2, and positive signal for C and CH_3.

Spectra of 2-(4-methoxyphenyl)-1H-imidazole-4-carbaldehyde (2b)

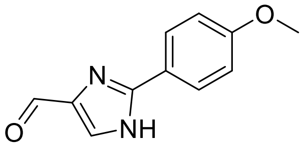

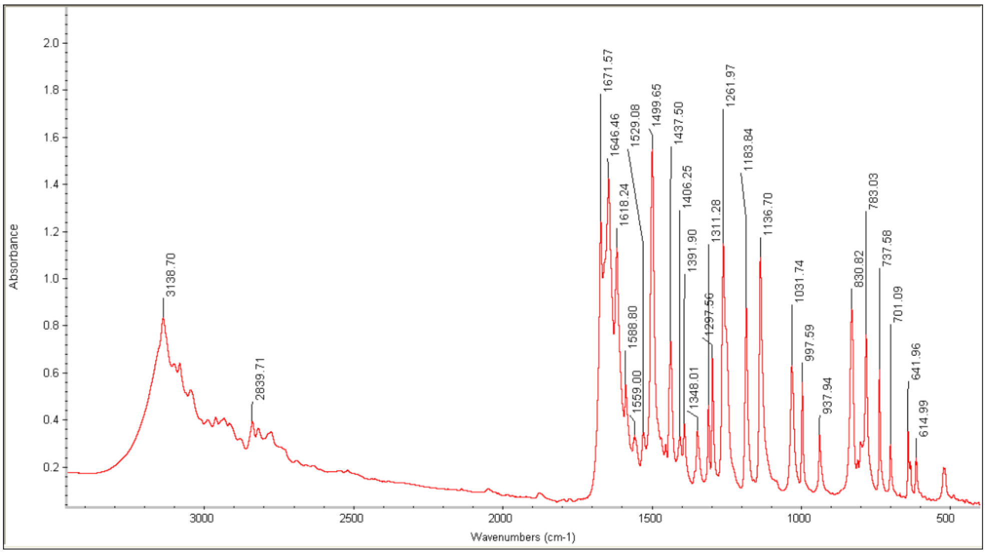

Figure 27. IR spectrum of **2b** in KBr pellets.

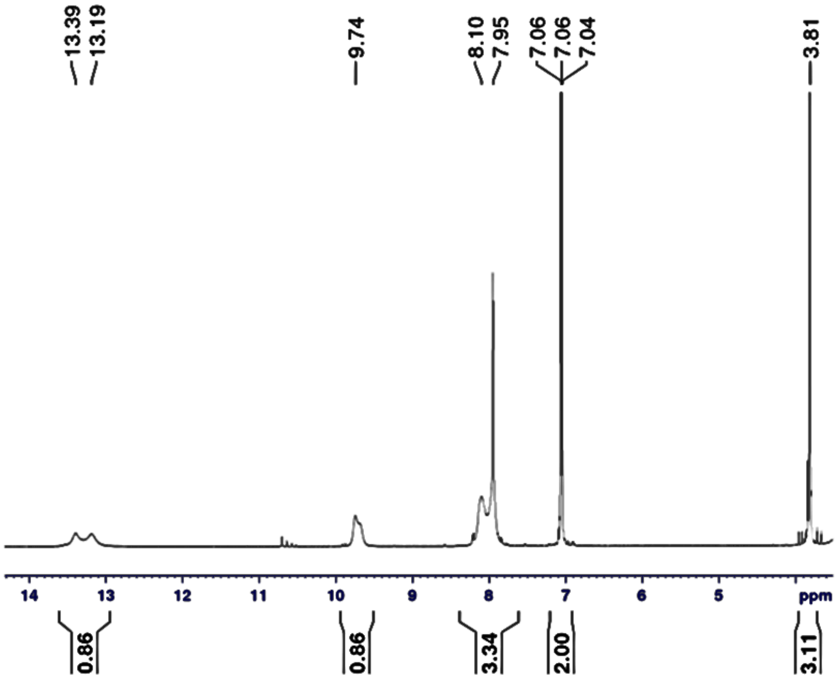

Figure 28. NMR (DMSO-d_6) of **2b** at 298 K.

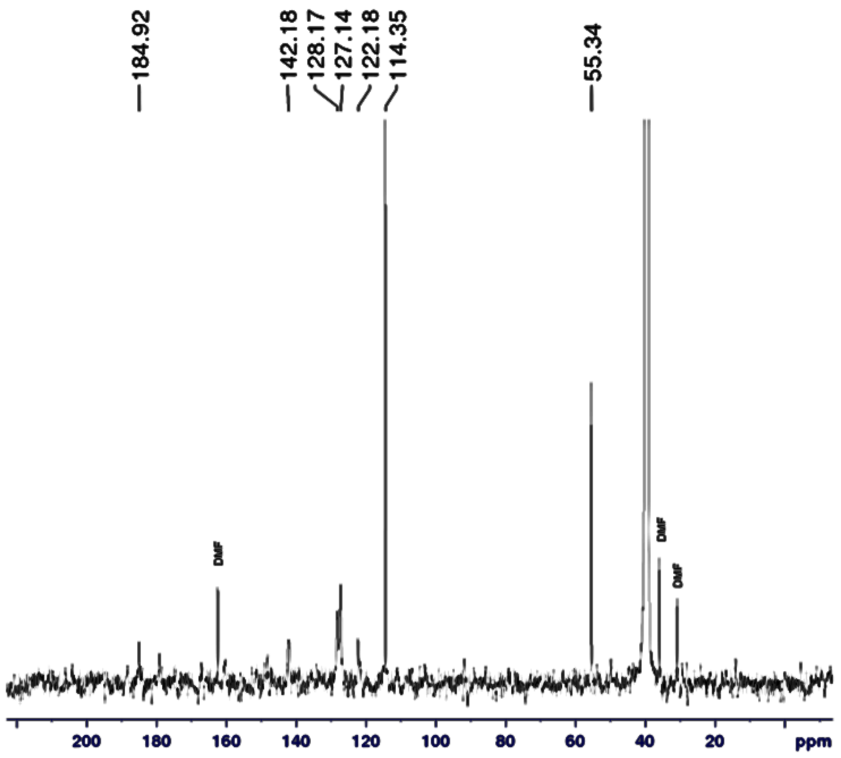

Figure 29. ^{13}C NMR (DMSO-d_6) of **2b** at 298 K (125MHz; NS 1024).

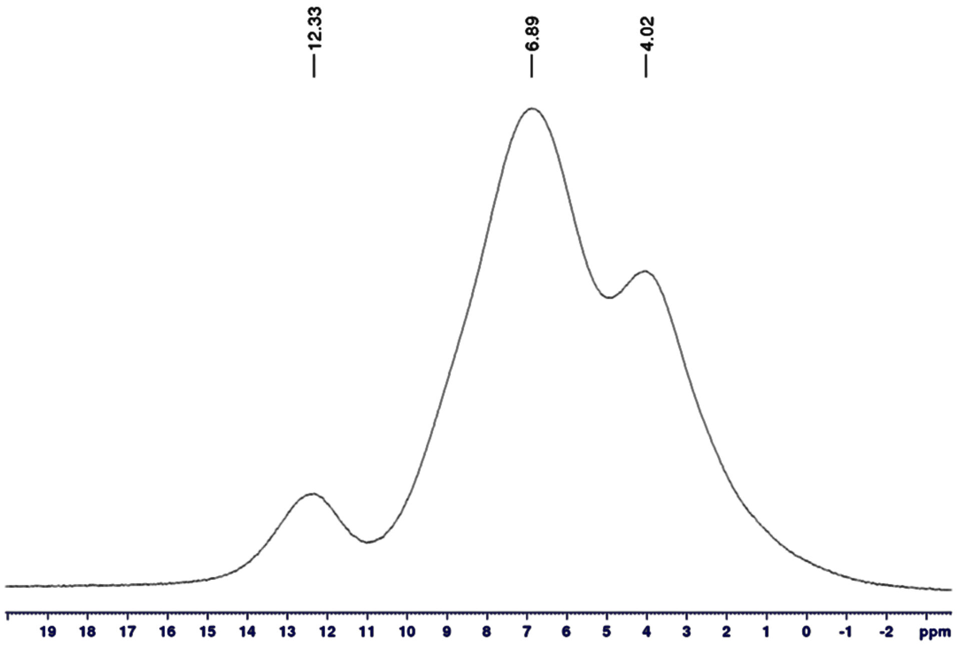

Figure 30. ^{1}H MAS NMR (solid state 850 MHz, 25 kHz) of **2b.**

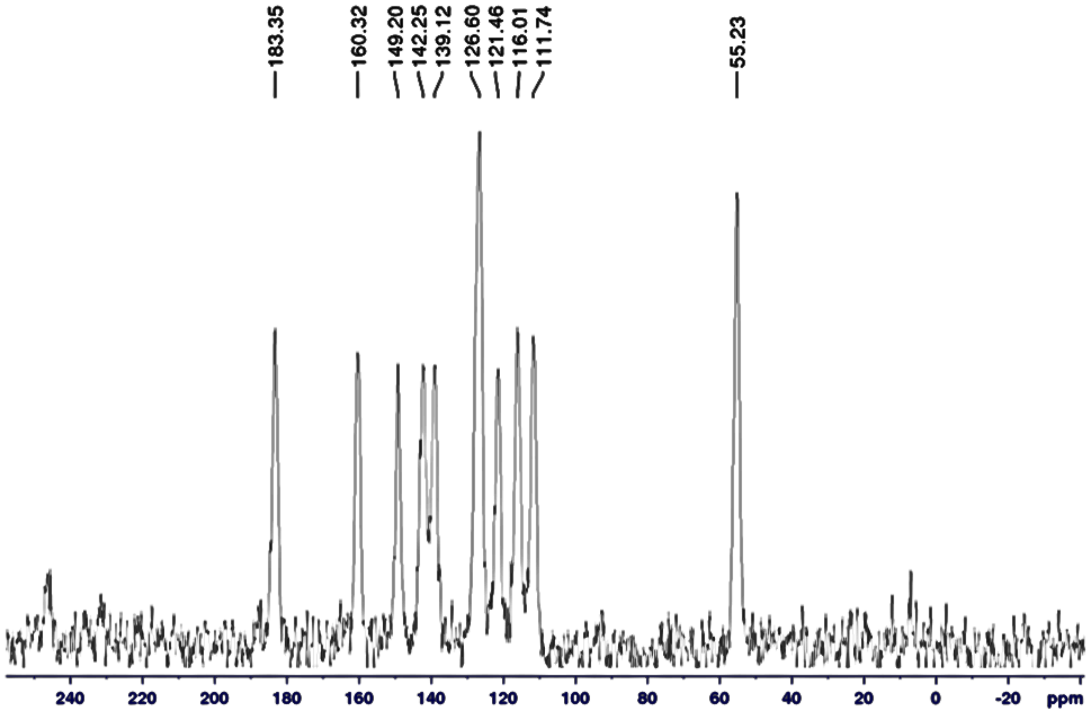

Figure 31. ^{13}C CP-MAS NMR (125 MHz, 15 kHz) of **2b.**

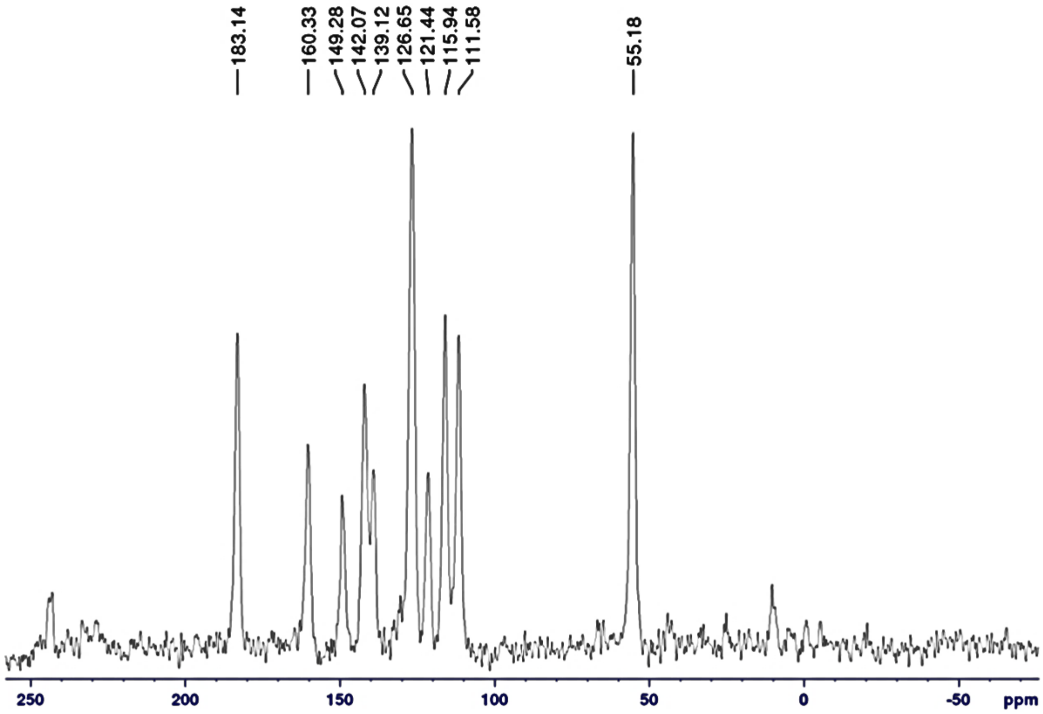

Figure 32. ^{13}C CP-MAS NMR (212.5 MHz, 25 kHz) of **2b.**

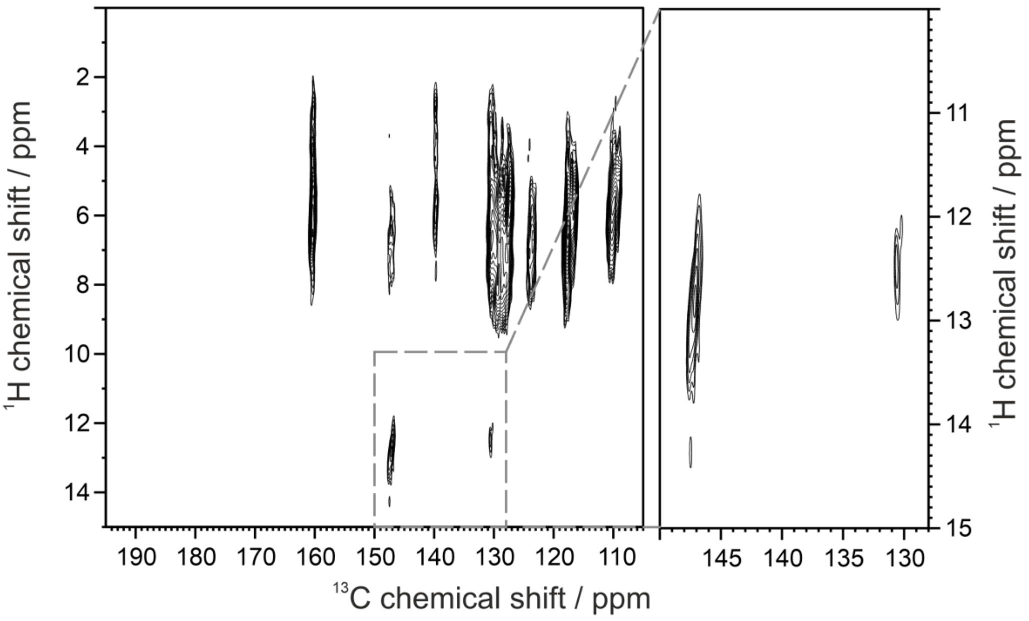

Figure 33. ^{1}H-^{13}C CP-MAS correlation spectrum (212.5 MHz, 25 kHz) of compound **2b**. Right block shows magnified view of the selected area in the correlation spectrum.

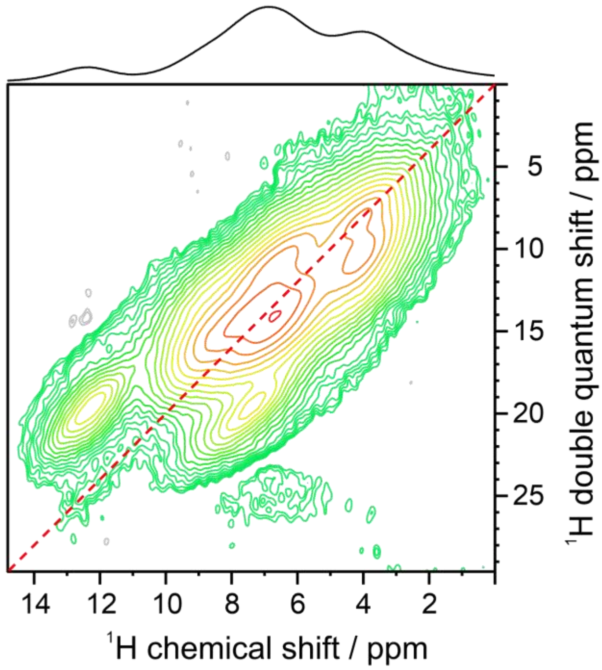

Figure 34. Double quantum-single quantum correlation spectrum of **2b** recorded at 25 kHz MAS, and 1 rotor periods double-quantum recoupling using the BABA-xy16 pulse sequence.

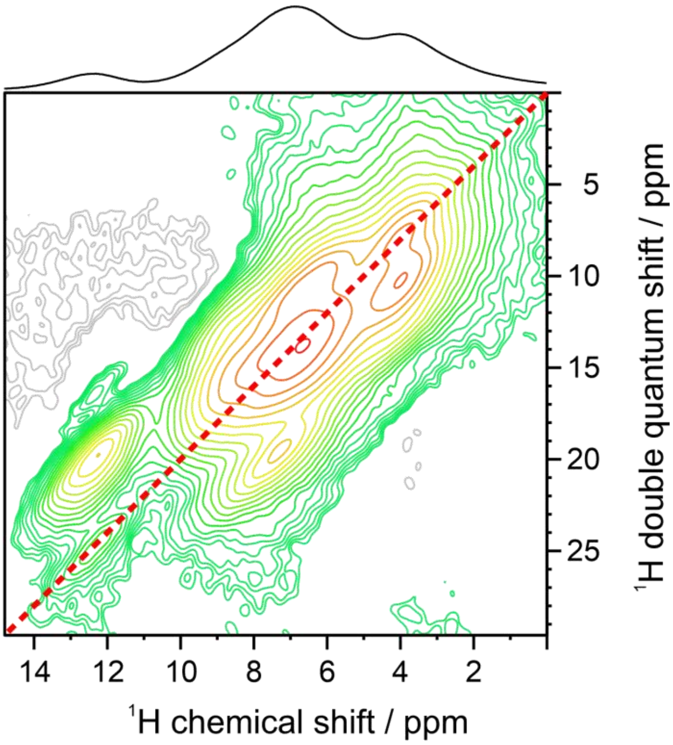

Figure 35. Double quantum-single quantum correlation spectrum of **2b** recorded at 25 kHz MAS, and 4 rotor periods double-quantum recoupling using the BABA-xy16 pulse sequence.

Spectra of 4-(4-formyl-1H-imidazol-2-yl)benzonitrile (2c)

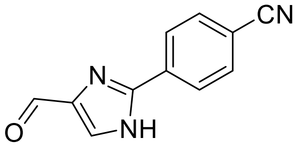

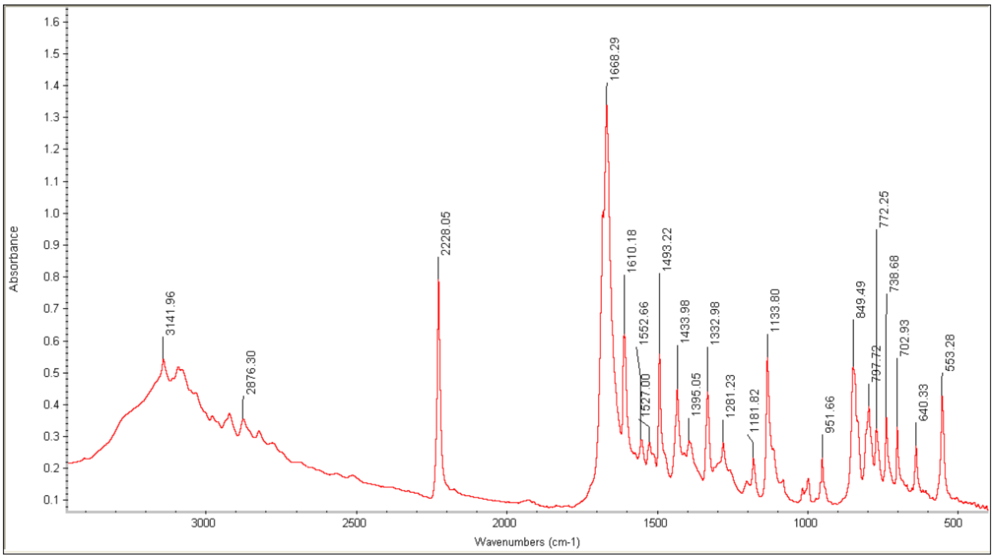

Figure 36. IR spectrum of **2c** in KBr pellets.

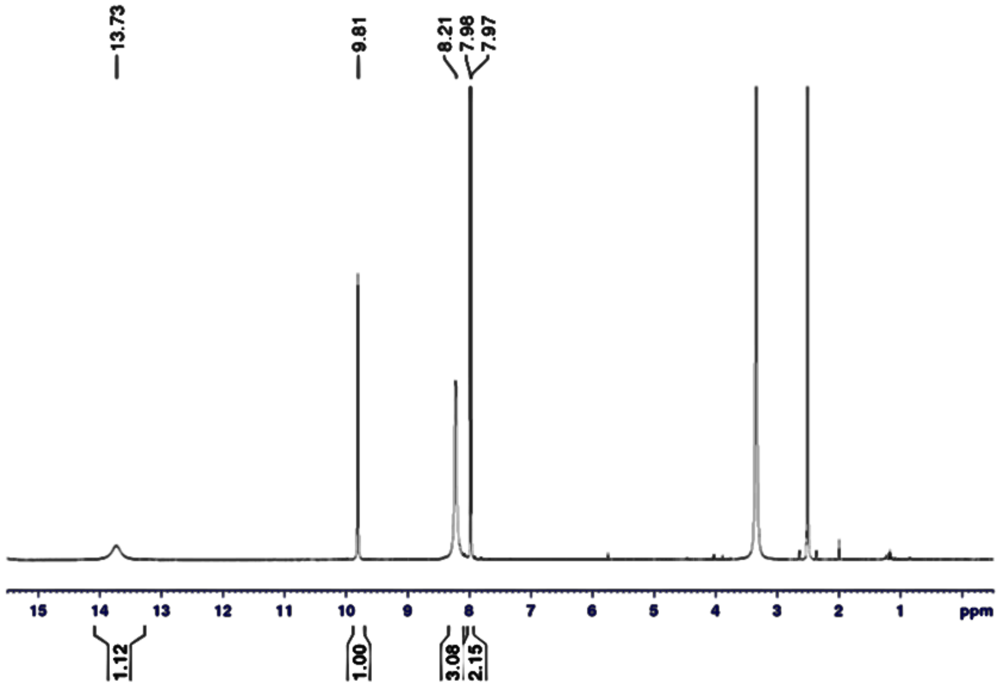

Figure 37. ^{1}H NMR (DMSO-d_6) of **2c** at 298 K.

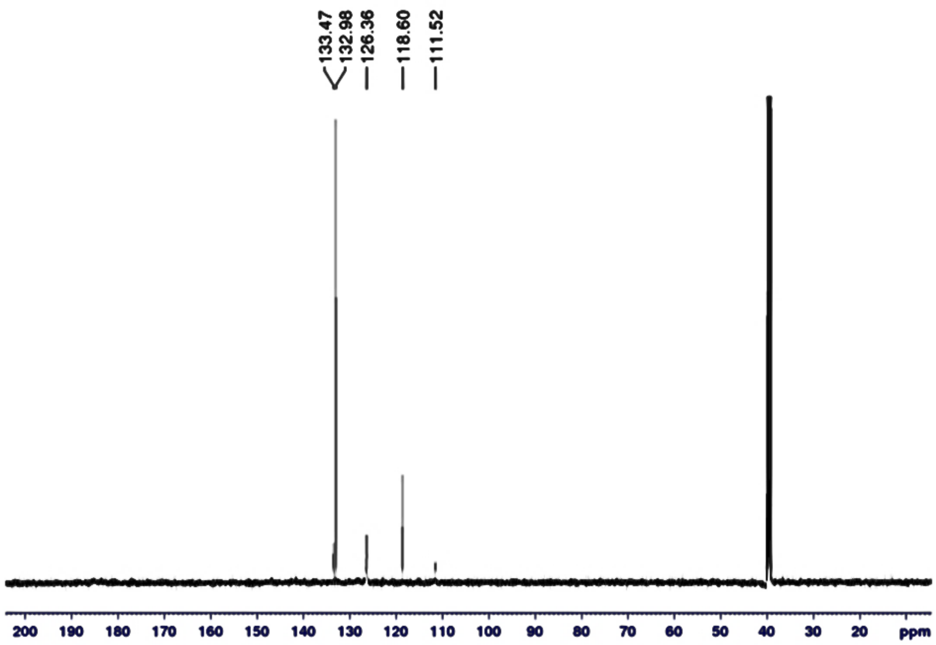

Figure 38. ^{13}C NMR (DMSO-d_6, 125 MHz) of **2c**.

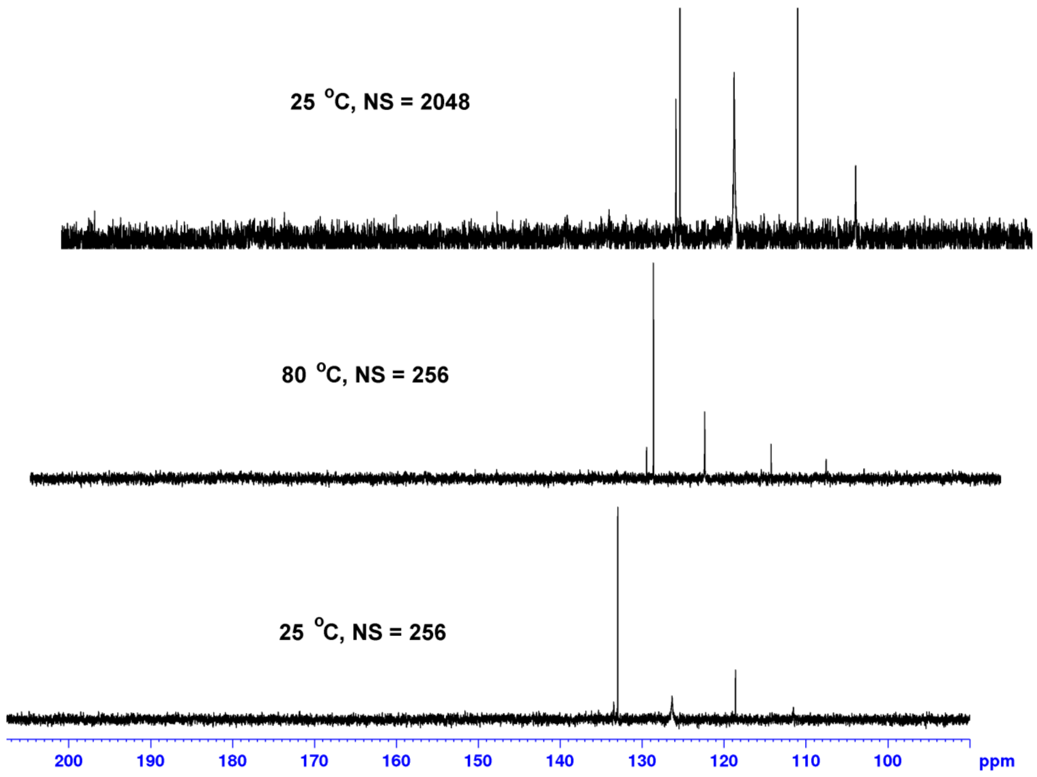

Figure 39. ^{13}C NMR (DMSO-d_6) of **2c** at two different temperatures (25 and 80 °C) and different scan number (NS) 256 and 2048.

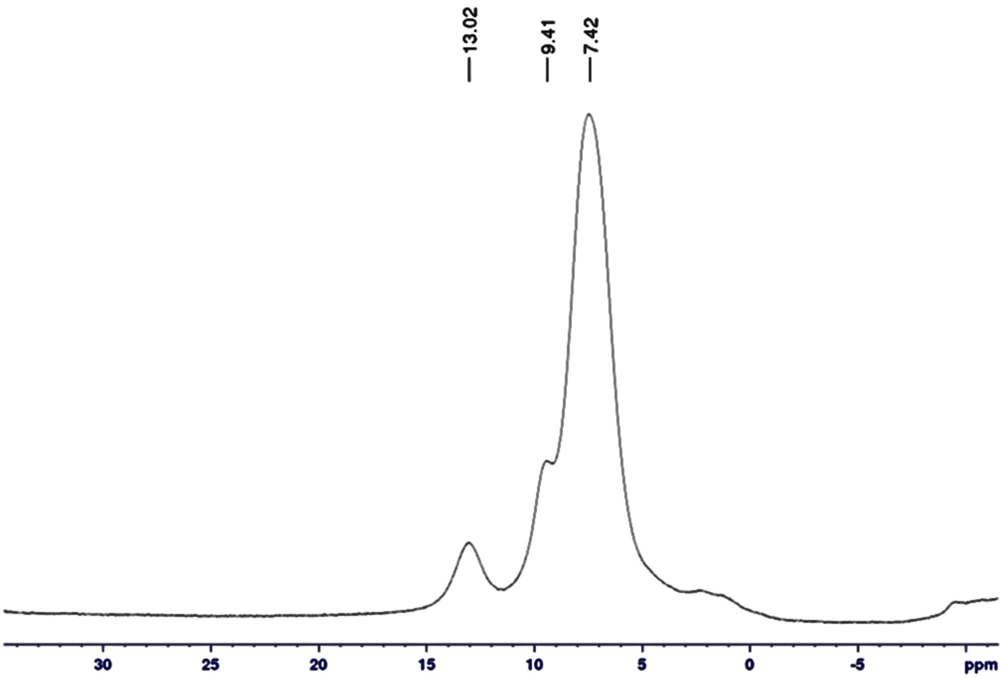

Figure 40. ^{1}H MAS NMR (850 MHz, 29 kHz) of **2c**.

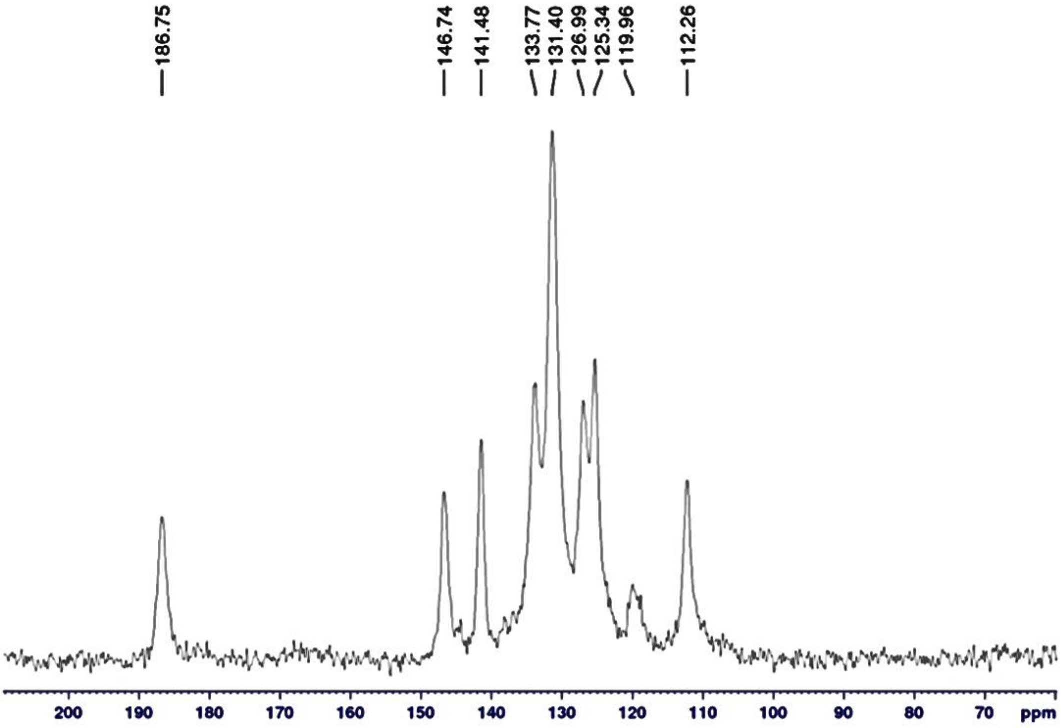

Figure 41. ^{13}C CP-MAS NMR (125 MHz, 10kHz) of **2c**.

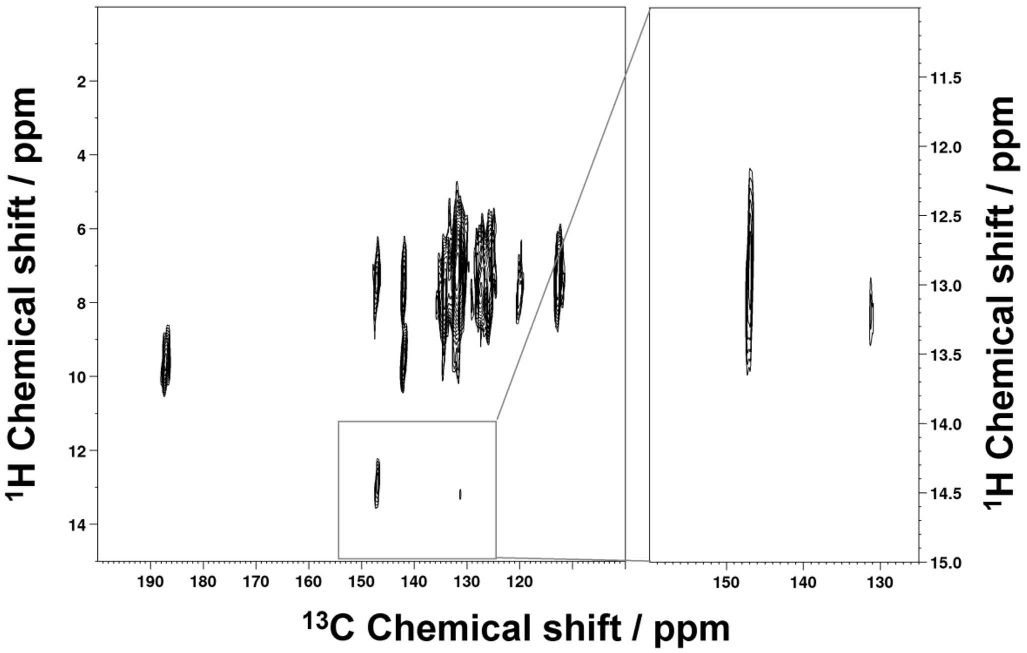

Figure 42. ^{1}H-^{13}C CP-MAS correlation spectrum (212.5 MHz, 25 kHz) of compound **2c**. Right block shows magnified view of the selected area in the correlation spectrum.

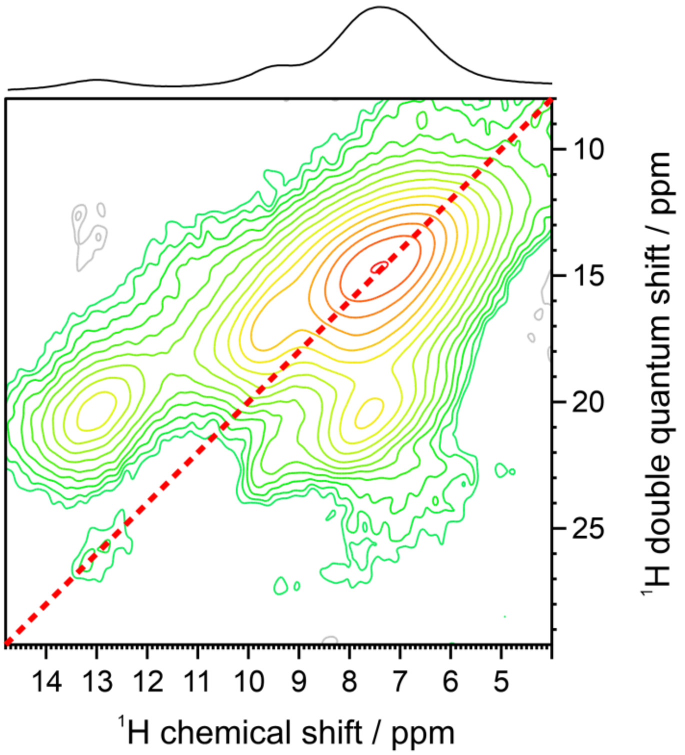

Figure 43. Double quantum-single quantum correlation spectrum of **2c** recorded at 25 kHz MAS, and 1 rotor periods double-quantum recoupling using the BABA-xy16 pulse sequence.

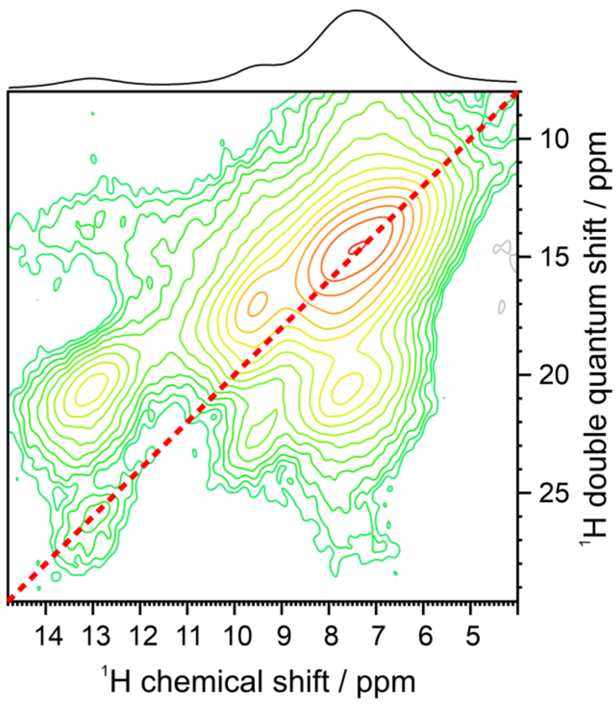

Figure 44. Double quantum-single quantum correlation spectrum of **2c** recorded at 25 kHz MAS, and 2 rotor periods double-quantum recoupling using the BABA-xy16 pulse sequence.

Spectra of 2-(4-hydroxyphenyl)-1H-imidazole-4-carbaldehyde (2d)

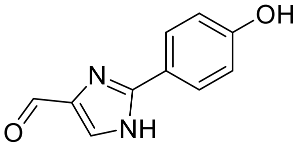

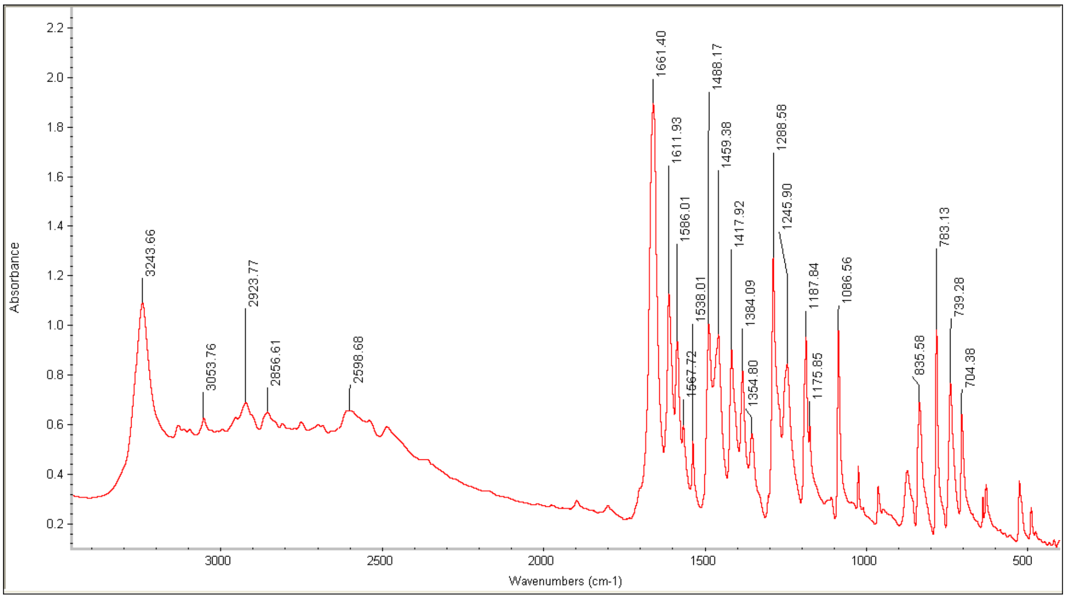

Figure 45. IR spectrum of **2d** in KBr pellets.

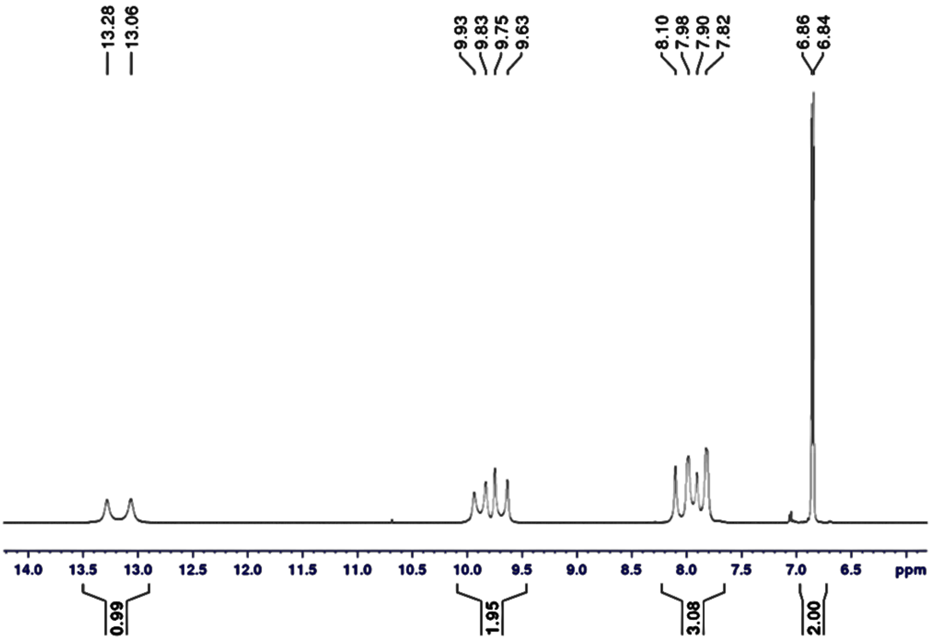

Figure 46. ^{1}H NMR (DMSO-d_6) of **2d** at 298 K (500 MHz, 25mM).

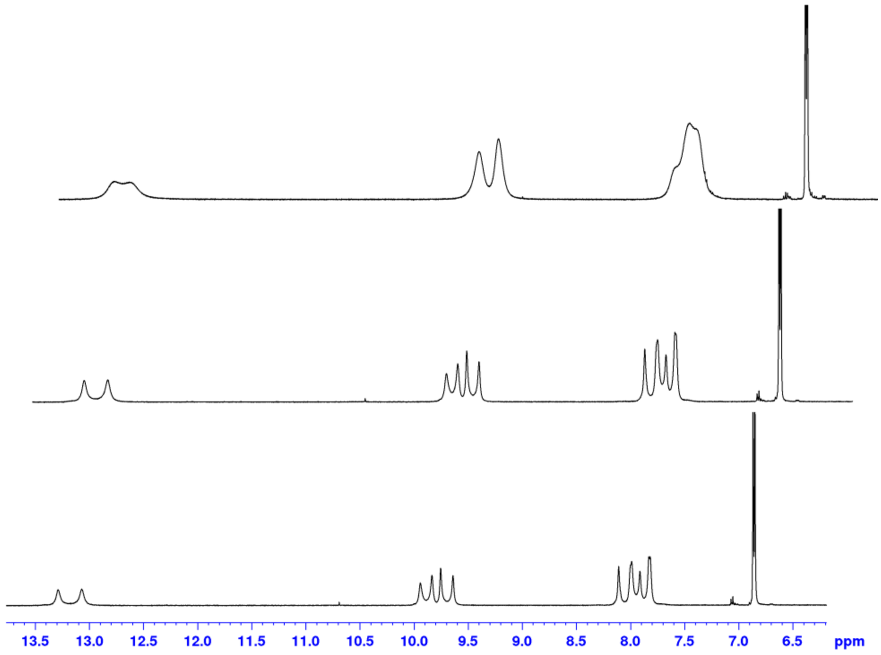

Figure 47. Concentration dependent ^{1}H NMR (DMSO-d_6) spectra of **2d** at 298 K (from bottom to top 13mM, 25mM and > 50 mM).

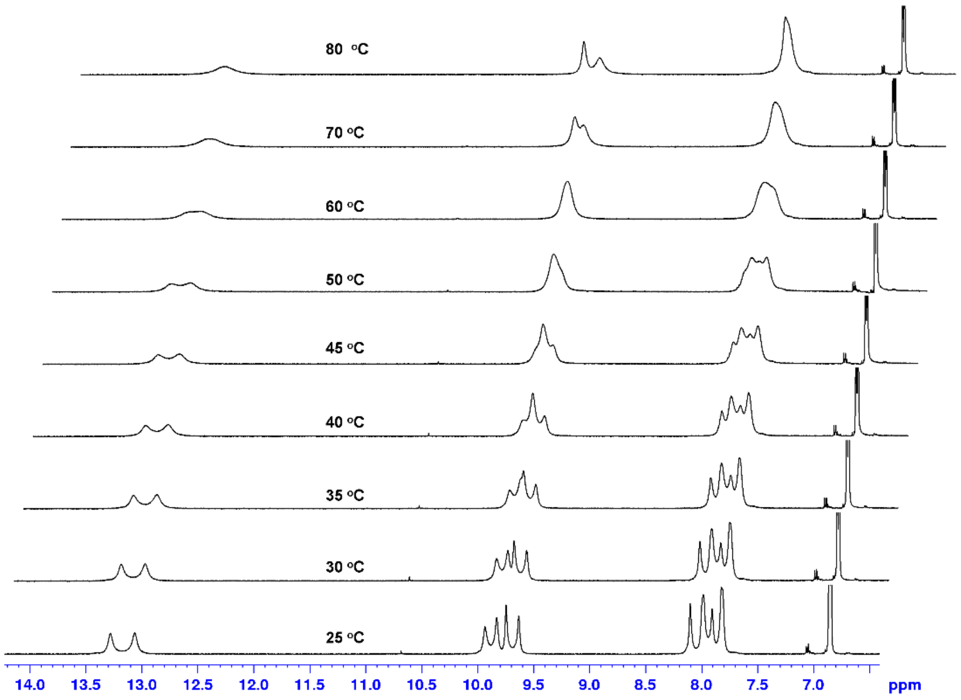

Figure 48. Temperature dependent ^{1}H NMR (DMSO-d_6) spectra of **2d** with concentration of 25mM.

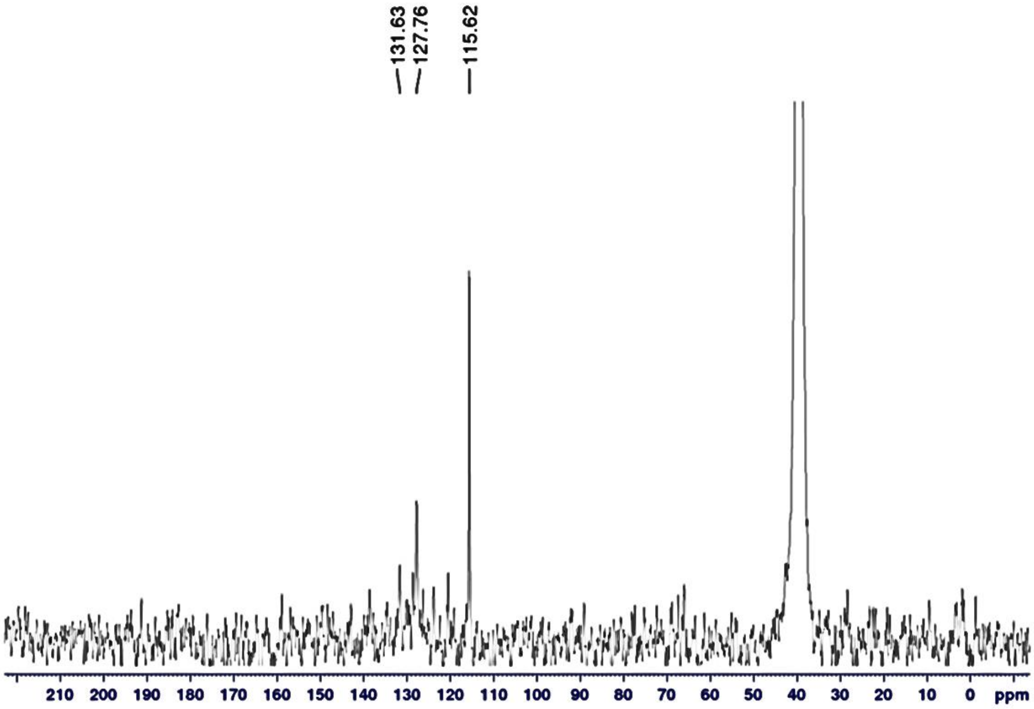

Figure 49. ^{13}C NMR (DMSO-d_6) spectra of **2d**; (125 MHz, NS 1024).

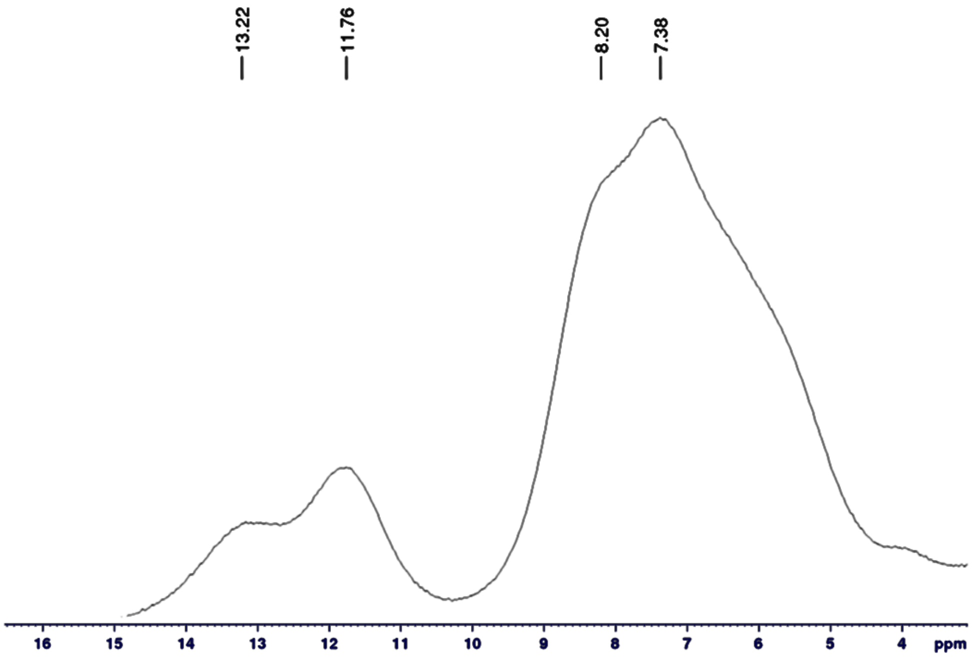

Figure 50. ^{1}H MAS NMR (850 MHz, 29 kHz) of **2d**.

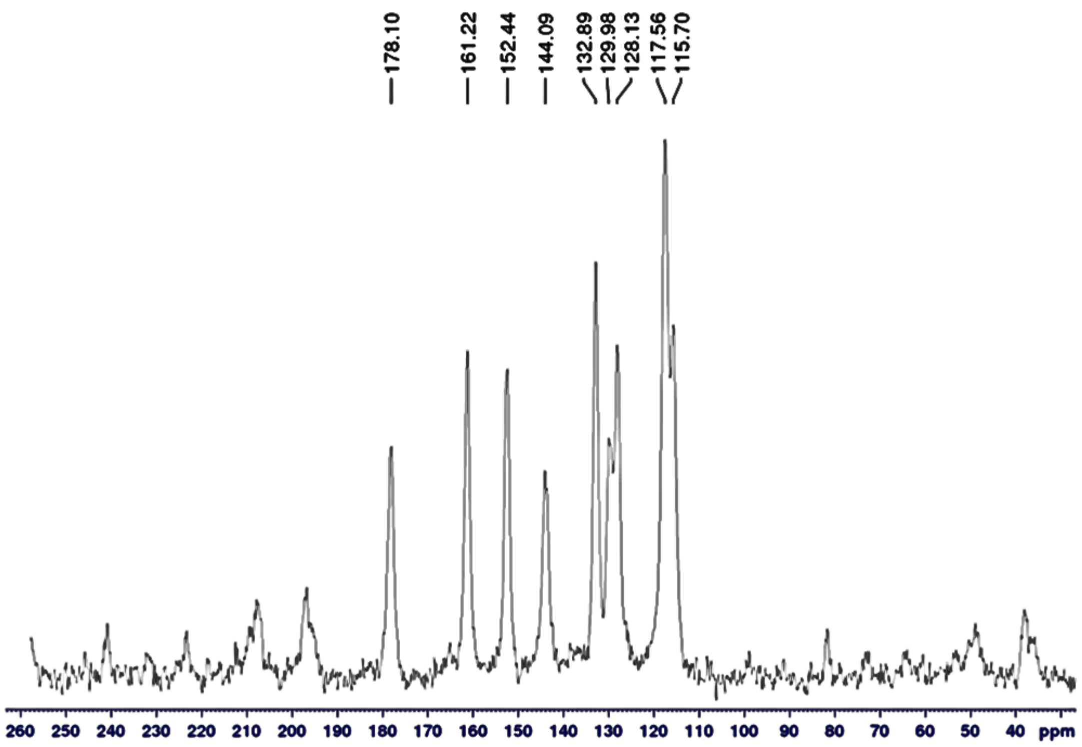

Figure 51. ^{13}C CP-MAS NMR (125 MHz, 10 kHz) of **2d**.

Spectra of 2-methyl-1H-imidazole-4-carbaldehyde

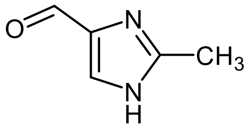

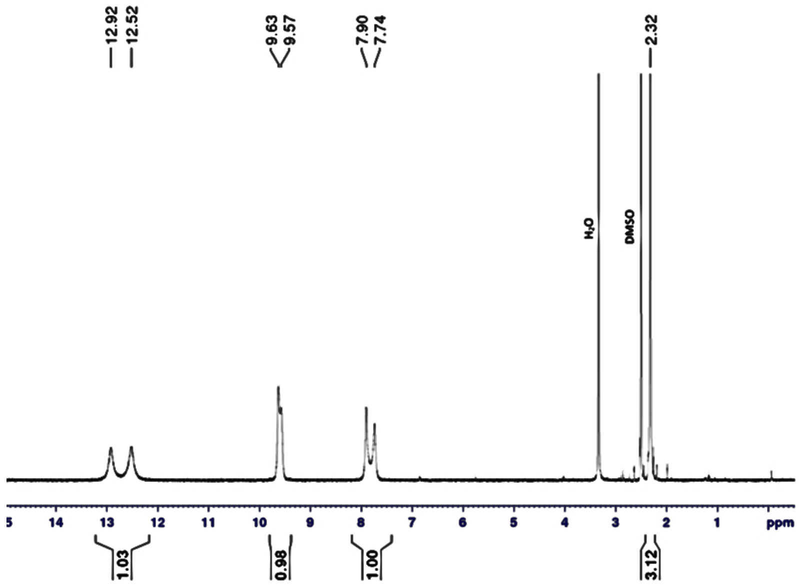

Figure 52. ^{1}H NMR (DMSO-d_6) at 298 K (500 MHz).

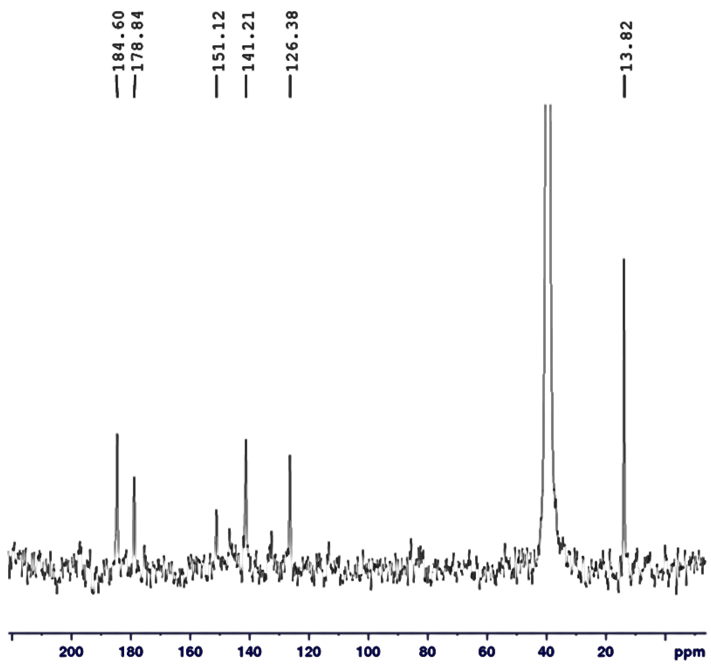

Figure 53. ^{13}C NMR (DMSO-d_6) at 298 K (125 MHz, 512 NS).

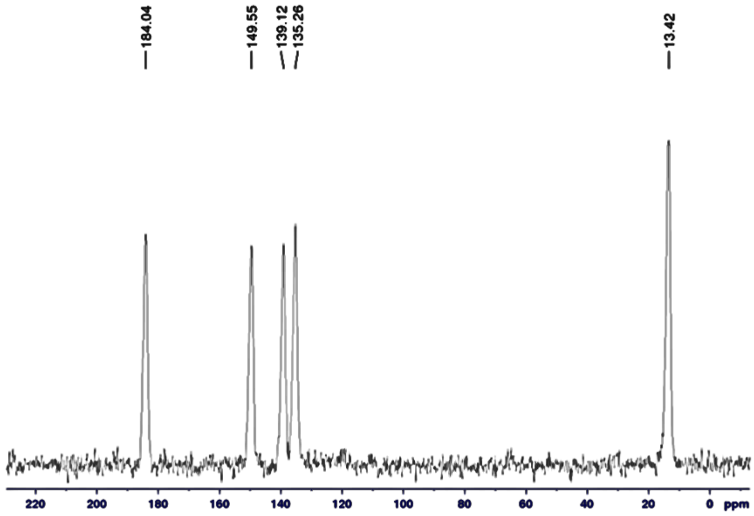

Figure 54. CP-MAS NMR (125 MHz, 15kHz).

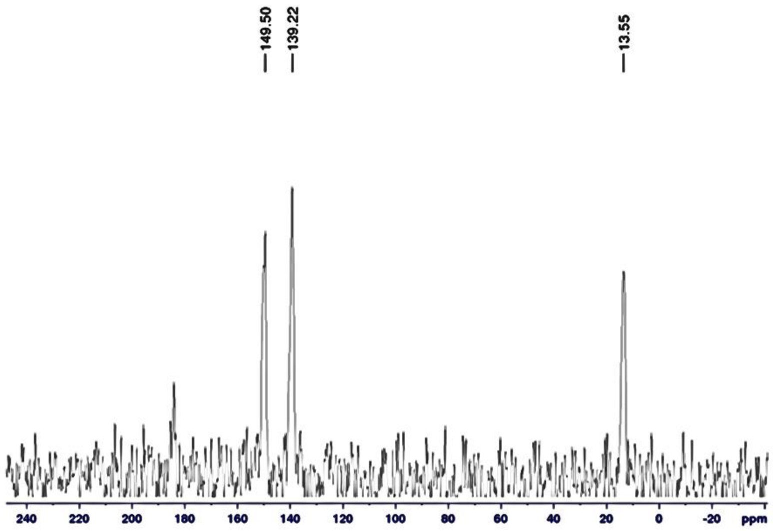

Figure 55. ^{13}C CPPI spectrum (125 MHz, 150 kHz); null signal for CH, negative signal for CH_2, and positive signal for C and CH_3.

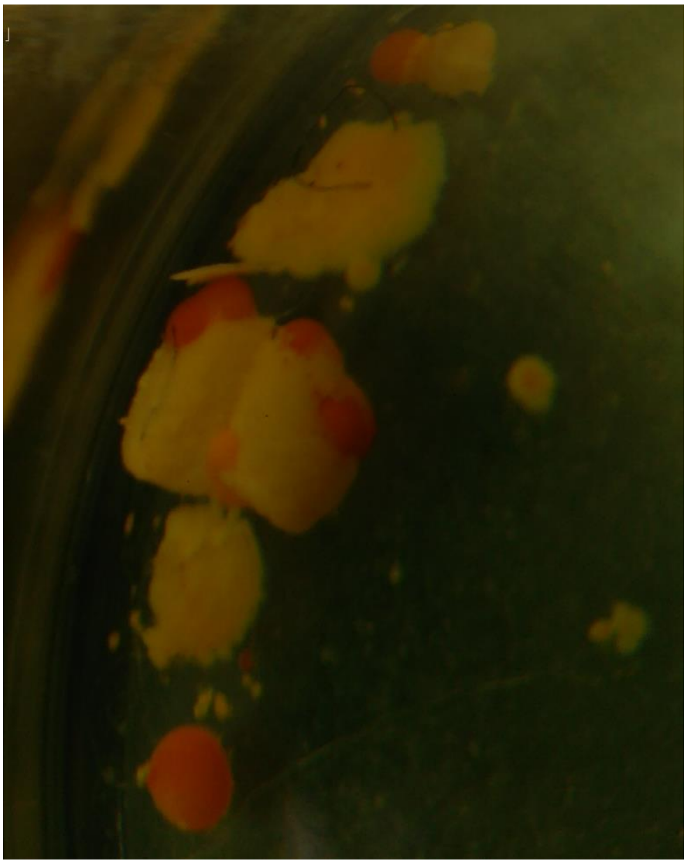

Figure 56. Microscope image of the different crystalline phases of **2c** .
